# Oral Egg-Derived Protein and Peptide Supplementation for Health Outcomes in Adults: Systematic Review and Meta-Analysis

**DOI:** 10.3390/nu18071054

**Published:** 2026-03-26

**Authors:** Eun Jeong Gong, Chang Seok Bang, Jae Jun Lee, Yong Seok Shin

**Affiliations:** 1 Department of Internal Medicine, Hallym University College of Medicine, Chuncheon 24253, Republic of Korea; 2 Institute for Liver and Digestive Diseases, Hallym University, Chuncheon 24253, Republic of Korea; 3 Institute of New Frontier Research, Hallym University College of Medicine, Chuncheon 24253, Republic of Korea; 4 Department of Anesthesiology and Pain Medicine, Hallym University College of Medicine, Chuncheon 24253, Republic of Korea

**Keywords:** egg protein, egg white, dialysis, albumin, bioactive peptides

## Abstract

**Background and Aims:** Egg-derived proteins and peptides have been investigated for various health outcomes, yet no comprehensive meta-analysis has synthesized this evidence to guide clinical practice. This study aimed to evaluate the efficacy of oral egg-derived protein and peptide supplementation on health outcomes, including serum albumin and phosphorus in dialysis patients, and visceral fat area, blood pressure, muscle protein synthesis, and cognitive function in adults. **Methods:** PubMed, Embase, Cochrane Library, and Web of Science were searched through January 2026 for RCTs. Random-effect meta-analyses, sensitivity analyses, and publication bias assessments were performed. Risk of bias was evaluated using the RoB 2 tool. Evidence certainty was evaluated using GRADE. **Results:** Thirty RCTs (n = 1938) were included. In dialysis patients, egg white supplementation significantly increased serum albumin (MD: +0.42 g/dL [95% CI: 0.12–0.72]; *I*^2^ = 82.4%; four RCTs; GRADE: very low) and decreased serum phosphorus (MD: −2.04 mg/dL [−2.50, −1.58]; *I*^2^ = 22%; two RCTs; GRADE: low). Leave-one-out sensitivity analysis showed consistency. Lactic-fermented egg white peptide reduced the visceral fat area (MD: −11.6 cm^2^ [−18.5, −4.8]; two RCTs; GRADE: very low). NWT-03 egg protein hydrolysate showed no significant effect on blood pressure (MD: +0.5 mmHg [−1.8, +2.7]; two RCTs). Publication bias was not detected. **Conclusions:** Egg-derived protein supplementation provides clinical benefits in dialysis patients with hypoalbuminemia, but evidence is lacking supporting its routine use in healthy adults or other clinical populations.

## 1. Introduction

Protein-energy wasting is a prevalent and prognostically significant complication in patients undergoing maintenance dialysis [1], with hypoalbuminemia (serum albumin < 3.8 g/dL) serving as one of the strongest independent predictors of mortality in this population [2,3].

Among the various protein sources investigated for clinical supplementation, egg-derived proteins occupy a unique position owing to their high biological value, complete amino acid profile, and favorable digestibility (protein digestibility-corrected amino acid score [PDCAAS] = 1.0) [4,5]. Egg white, consisting primarily of ovalbumin (54%), ovotransferrin (12%), ovomucoid (11%), and lysozyme (3.5%) [6], provides a highly bioavailable protein source that has been specifically studied in malnourished dialysis patients as a strategy to improve nutritional status and reduce serum phosphorus levels without proportionally increasing phosphate burden [7,8,9,10].

Beyond their role as a macronutrient source, egg proteins have been identified as precursors to bioactive peptides, with physiological functions extending well beyond basic nutrition [11]. Enzymatic hydrolysis of ovalbumin yields peptides with angiotensin-converting enzyme (ACE) inhibitory activity, including the tripeptide IRW (Ile-Arg-Trp) and longer sequences such as ovokinin and ovokinin (2–7) [12,13]. Commercial egg protein hydrolysates, such as NWT-03 (Newtricious R&D, Venlo, The Netherlands) [14] and lactic-fermented egg white (LAFEW; Kewpie Corporation, Tokyo, Japan) [15], have been developed and marketed for their cardiovascular and metabolic health benefits, respectively. Additionally, Fortetropin (MYOS Corporation, Cedar Knolls, NJ, USA), a proteo-lipid complex derived from fertilized egg yolk, has been investigated for its potential to suppress circulating myostatin levels and promote muscle protein synthesis. The global market for egg-derived functional proteins has grown rapidly, particularly in East Asia, where the consumer demand for protein-enriched health products has outpaced the available clinical evidence.

Despite growing commercial interest and numerous randomized controlled trials (RCTs), the evidence base for egg-derived protein and peptide supplementation remains fragmented. Clinical trials have examined diverse populations, including dialysis patients, elderly individuals with sarcopenia, athletes, and adults with metabolic syndrome or mild hypertension, using heterogeneous interventions ranging from intact egg white protein to specific bioactive peptide formulations [7,8,9,10,14,15,16]. This fragmentation has precluded pooled analysis and has contributed to a disconnect between marketing claims and the strength of underlying evidence.

The existing systematic reviews (SRs) and meta-analyses in this field have addressed related but distinct questions, none of which specifically focused on egg-derived protein or peptide supplementation as the primary intervention (Table S1). Kolahdouz-Mohammadi et al. [17] examined the effect of whole-egg consumption on blood pressure (BP), while Morton et al. [18] investigated protein supplementation broadly for resistance training-induced muscle gains. The Cochrane review by Mah et al. [19] evaluated oral protein supplements in chronic kidney disease (CKD) but did not conduct egg-specific subgroup analyses. Network meta-analyses by Liao et al. [20] compared protein sources for muscle outcomes but did not separately analyze egg protein. Reviews of food-derived antihypertensive peptides [21,22] have been dominated by milk-derived peptides, with egg-derived peptides comprising only a minor fraction of included studies. To date, no SR or meta-analysis has specifically examined the effects of oral egg-derived protein or peptide supplementation on health outcomes in adults.

This gap is particularly significant given the expanding market for egg protein supplements, especially in Asia, where the evidence–practice disparity is most pronounced. In South Korea and Japan, egg white protein supplements and fermented egg white products are widely marketed with health claims that have not been subjected to rigorous evidence synthesis. Conversely, the European evidence base for NWT-03 hydrolysate, while methodologically rigorous, has not demonstrated consistent clinical benefits across trials [14,23,24,25].

Therefore, we conducted a SR and meta-analysis of all available RCTs to evaluate the efficacy of oral egg-derived protein and peptide supplementation on health outcomes in adults. We aimed to (1) quantify the effects of egg white protein supplementation on serum albumin and phosphorus in dialysis patients; (2) assess the effects of egg-derived bioactive peptides on cardiovascular, metabolic, and cognitive outcomes; (3) evaluate the overall quality and certainty of the evidence and (4) identify the evidence–practice gap to inform clinical guidelines and future research priorities.

## 2. Methods

The detailed methodology is described in Supplementary Files S1–S3 [26,27,28,29,30,31,32,33,34,35]. A brief summary is provided below.

This systematic review and meta-analysis was conducted following the PRISMA 2020 guidelines [26] and was registered prospectively in PROSPERO (CRD420261295226). PubMed/MEDLINE, Embase-OVID, Cochrane CENTRAL, and Web of Science were searched from inception through 31 January 2026, combining MeSH terms and free-text keywords for egg-derived protein/peptide interventions and randomized controlled trial design filters. Trial registries (ClinicalTrials.gov, ICTRP) were also searched. No language or date restrictions were applied. Eligible interventions included intact egg white protein (powder, liquid, or cooked egg white), egg protein hydrolysates (e.g., NWT-03 ovalbumin hydrolysate), lactic-acid-fermented egg white (LAFEW), egg white extract, Fortetropin (egg yolk-derived proteo-lipid complex), and other specific egg-derived bioactive peptides. Studies were included if they were RCTs (parallel or crossover) enrolling adults (≥18 years) comparing egg-derived supplements to placebo, no treatment, or usual care. Primary outcomes were serum albumin, serum phosphorus, systolic and diastolic blood pressure, and body composition parameters. Secondary outcomes included lipid profiles, muscle protein synthesis, cognitive function, and adverse events.

Data were extracted independently by two reviewers (CSB and EJG). Risk of bias was assessed using the Cochrane RoB 2 tool [28] across five domains: randomization process, deviations from intended interventions, missing outcome data, measurement of the outcome, and selection of the reported result. Random-effects meta-analyses using the DerSimonian–Laird estimator [29] were performed when at least two studies reported comparable outcomes. Heterogeneity was assessed using the *I*^2^ statistic [30]. Leave-one-out sensitivity analyses, publication bias assessment (Egger’s and Begg’s tests) [33,34], and GRADE certainty assessment were conducted. All analyses were performed using R (version 4.3.2) with the meta and metafor packages.

## 3. Results

### 3.1. Study Selection

The systematic search across PubMed/MEDLINE, Embase-OVID, Cochrane CENTRAL, Web of Science, and registry search identified 5770 records after deduplication. Title and abstract screening excluded 5350 records, and 420 full-text articles were assessed for eligibility. Of these, 390 were excluded for the following reasons: SR (n = 6), narrative review (n = 20), and insufficient data (n = 364). Ultimately, 30 publications [7,8,9,10,14,15,16,23,24,25,36,37,38,39,40,41,42,43,44,45,46,47,48,49,50,51,52,53,54,55] reporting on 26 unique trial cohorts were included in the qualitative synthesis, and data from four meta-analyzable outcomes were pooled quantitatively (Figure S1).

Among the 30 publications, three pairs of confirmed companion reports and one pair of related publications were identified: Nijssen et al. [23] and Gravesteijn et al. [24] reported arterial stiffness and cognitive outcomes, respectively, from the same trial cohort (NCT02561663; n = 76); Adams et al. reported endothelial function [25] and cognitive outcomes [36] from a single trial (NCT04831203; n = 44); and Matsuoka et al. 2017a [15] and 2017b [16] reported cholesterol and visceral fat outcomes from overlapping but distinct participant samples. Azmandian et al. 2022 [9] and Azmandian et al. 2025 [37] were initially considered companion reports, as both originated from the same research program at Kerman University of Medical Sciences and employed an identical egg white intervention protocol. However, the two studies used different inclusion criteria (serum phosphorus ≥ 5.5 mg/dL for the 2022 study; hemoglobin < 12 g/dL requiring erythropoietin for the 2025 study) and enrolled different sample sizes (n = 150 and n = 107, respectively), suggesting these may represent related but distinct trial cohorts. These linked publications were treated conservatively in the meta-analysis to avoid potential double-counting of participants.

### 3.2. Study Characteristics

The 30 included publications (26 unique trials) enrolled a total of 1938 participants across 12 countries. Study duration ranged from acute single-dose designs (5–6 h) to 36 weeks. The included trials could be broadly categorized into five intervention types based on the egg-derived product studied.

First, five trials evaluated intact egg white protein supplementation in maintenance dialysis patients: González-Espinoza et al. 2005 [7] (n = 28 randomized, continuous ambulatory peritoneal dialysis [CAPD], Mexico, 6 months), Guida et al. 2019 [8] (n = 23, hemodialysis [HD] with hyperphosphatemia, Italy, 3 months), Azmandian et al. 2022 [9] (n = 150 randomized, 128 analyzed; HD, Iran, 8 weeks), and Javadian et al. 2024 [10] (n = 98, HD, Iran, 8 weeks) and Azmandian et al. 2025 [37] (n = 107, HD, Iran, 8 weeks).

Second, four trials evaluated egg-derived protein for metabolic and weight management outcomes. Three trials evaluated LAFEW in Japanese adults with specific metabolic conditions: Matsuoka et al. 2017a [15] (n = 88, mildly hypercholesterolemic men, 8 weeks), Matsuoka et al. 2017b [16] (n = 37, obese subjects, 12 weeks), and Matsuoka et al. 2019 [38] (n = 22, overweight males, 8 weeks). Additionally, Brun et al. 2018 [39] (n = 22 + 337, obese adults, France, 2 months/18 months) evaluated purified egg protein (Ovamine^®^) for weight management.

Third, seven publications from five unique trials investigated the egg protein hydrolysate NWT-03 in European adults with mild hypertension, metabolic syndrome, or overweight/obesity: Plat et al. 2017 [14] (n = 92, dose-finding, crossover, The Netherlands, 4 weeks/period), Lucey et al. 2019 [40] (n = 75, crossover, mildly elevated BP, Ireland), Plat et al. 2019 [41] (n = 40, acute crossover, overweight/obese with impaired glucose tolerance), Nijssen et al. 2023 [23] (n = 76, metabolic syndrome, crossover, The Netherlands, 4 weeks/period), and Gravesteijn et al. 2023 [24] (n = 76, crossover, same cohort as Nijssen, cognitive outcomes), and Adams et al. 2025 [25] (n = 44, older adults with overweight/obesity, parallel, double-blind, The Netherlands, 36 weeks, endothelial function) [36] (n = 44, same cohort as Adams 2025, cognitive outcomes).

Fourth, ten studies examined egg protein in the context of exercise, MPS, or sarcopenia. These include Moore et al. 2009 [42] (n = 6, acute dose–response for MPS, Canada), Van Vliet et al. 2017 [52] (n = 10, acute crossover, whole eggs vs. egg whites, USA), and Fuchs et al. 2022 [46] (n = 45, acute, raw vs. boiled eggs, The Netherlands), Hida et al. 2012 [43] (n = 30, female collegiate athletes, Japan, 8 weeks), Kato et al. 2011 [44] (n = 30, healthy young adults, Japan, 5 weeks), Ullevig et al. 2022 [45] (n = 20, community-dwelling older Latina women, USA, pilot, 24 weeks), Bagheri et al. 2021 [47] (n = 30, trained young males, Iran, 12 weeks), Sharp et al. 2016 [53] (n = 37, resistance-trained college males, USA, 12 weeks), Evans et al. 2021 [54] (n = 20, older adults, USA, 21 days), and Lim et al. 2023 [55] (n = 24, healthy young men, Canada, 6 weeks including 2-week immobilization).

Fifth, four trials addressed miscellaneous populations and outcomes: Bhurayanontachai et al. 2016 [48] (n = 40, critically ill patients with respiratory failure, Thailand, ICU enteral feeding, 7 days), Mukmin et al. 2024 [49] (n = 46, malnourished elderly, Indonesia, 2 weeks), Oe et al. 2020 [50] (n = 19 + 74, athletes, Japan, 2 weeks), and Markus et al. 2010 [51] (n = 35, healthy adults, The Netherlands, acute stress paradigm). The characteristics of all included studies are detailed in Table 1.

### 3.3. Risk of Bias Assessment

The overall risk of bias was judged as low in 6 studies (20%), some concerns in 16 studies (53%), and high in 8 studies (27%) (Table S2; Figure S2).

At the domain level, bias arising from the randomization process (D1) was the most problematic domain, with 18 studies (60%) rated as having some concerns or high risk, primarily due to open-label designs in dialysis trials where allocation concealment was either absent or inadequately reported, and in exercise/muscle studies where blinding of food-form interventions (whole eggs vs. egg whites) was not feasible. Bias due to deviations from intended interventions (D2) was judged as having some concerns or high in 10 studies (33%), predominantly in open-label trials where participants’ knowledge of their intervention assignment may have influenced adherence and co-intervention behavior. Bias due to missing outcome data (D3) was generally well-managed, with 18 studies (60%) rated as low risk; however, two trials [39,45] had attrition rates exceeding 30%, raising concerns about potential attrition bias. Bias in measurement of the outcome (D4) was rated as low in the vast majority of studies (29 of 30; 97%); the dialysis studies measuring objective laboratory outcomes (serum albumin, phosphorus) and the exercise studies using isotope tracer kinetics, DXA, or ultrasonography were inherently less susceptible to measurement bias. Bias in selection of the reported results (D5) was judged as having some concerns or high in 20 studies (67%), primarily when pre-registered protocols were unavailable or when outcome reporting appeared selective.

Among the four studies contributing to the meta-analyzed albumin outcome, three [7,9,37] were rated as high risk of bias, predominantly due to lack of blinding in open-label designs with potential for performance bias, and one [8] was rated as some concerns. This concentration of high-risk studies within the albumin meta-analysis contributed to the GRADE downgrading for risk of bias.

The NWT-03 trials from the Maastricht research group [14,23,24,25,36,40,41] were consistently rated as low risk of bias, reflecting their rigorous double-blind, placebo-controlled designs with adequate allocation concealment and low attrition rates. The Japanese LAFEW trials [15,16,38] were judged as low risk or some concerns, with their double-blind, placebo-controlled parallel designs meeting most quality criteria despite industry sponsorship by the Kewpie Corporation.

Among the exercise and MPS studies, Lim et al. 2023 [55] was rated as low risk of bias due to its double-blind design with flavor-matched supplements, adequate randomization, zero attrition, and prospective trial registration. The remaining exercise studies [47,52,53,54] were rated as some concerns, primarily due to an inability to blind food-form interventions (Bagheri, Van Vliet) or the absence of prospective trial registration combined with industry sponsorship by the MYOS Corporation (Sharp, Evans).

### 3.4. Meta-Analysis Results

Quantitative meta-analysis was feasible for four outcomes where two or more RCTs with compatible interventions, populations, and outcome measures reported data amenable to pooling (Table S3).

### 3.5. Serum Albumin (Dialysis Patients)

**Figure 1:**
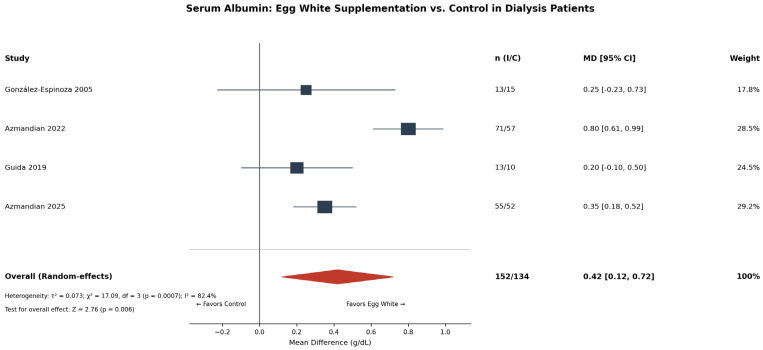
Forest plot of the effect of egg white protein supplementation on serum albumin in maintenance dialysis patients [7,8,9,37]; squares represent individual study effect sizes, with square size proportional to study weight; horizontal lines represent 95% confidence intervals. The diamond represents the pooled estimate, with diamond width indicating the 95% CI. The vertical solid line represents the null effect (MD = 0). n (I/C), number of participants in the intervention/control group; MD, mean difference; CI, confidence interval.

Four RCTs [7,8,9,37] evaluated the effect of egg white protein supplementation on serum albumin in maintenance dialysis patients. The pooled MD was +0.42 g/dL (95% CI: 0.12 to 0.72; *p* = 0.006), indicating a statistically significant increase in serum albumin with egg white supplementation (Figure 1). However, substantial heterogeneity was observed (*I*^2^ = 82.4%; τ^2^ = 0.07; Q = 17.0, *p* < 0.001). Individual study estimates ranged from +0.20 g/dL (Guida 2019 [8], rated as some concerns) to +0.80 g/dL (Azmandian 2022 [9], rated as high risk). The GRADE certainty was rated as very low, downgraded for risk of bias (three of four studies at high risk), inconsistency (*I*^2^ > 75%), and imprecision (wide CI).

### 3.6. Serum Phosphorus (Dialysis Patients)

**Figure 2:**
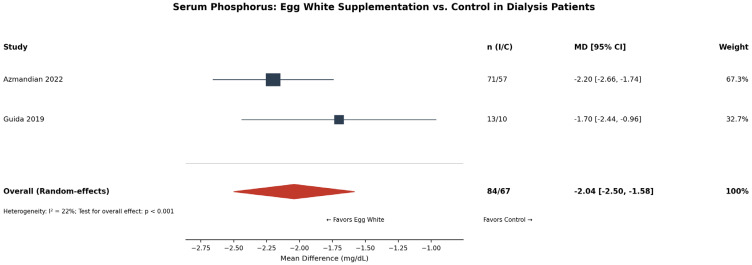
Forest plot of the effect of egg white-based dietary intervention on serum phosphorus in hemodialysis patients [8,9]; Squares represent individual study effect sizes, with square size proportional to study weight; horizontal lines represent 95% confidence intervals. The diamond represents the pooled estimate, with diamond width indicating the 95% CI. The vertical solid line represents the null effect (MD = 0). n (I/C), number of participants in the intervention/control group; MD, mean difference; CI, confidence interval.

Two RCTs [8,9] (total n = 151) evaluated the effect of egg white-based dietary interventions on serum phosphorus levels in hemodialysis patients. The pooled MD was −2.04 mg/dL (95% CI: −2.50 to −1.58; *I*^2^ = 22%), demonstrating a statistically significant reduction in serum phosphorus with egg white substitution (Figure 2). Both studies replaced high-phosphorus animal protein sources with egg whites in the diet of hyperphosphatemic dialysis patients. The low heterogeneity and consistent direction of effect across studies support the robustness of this finding. The GRADE certainty was rated as low, downgraded for risk of bias and the small number of studies.

### 3.7. VFA

**Figure 3:**
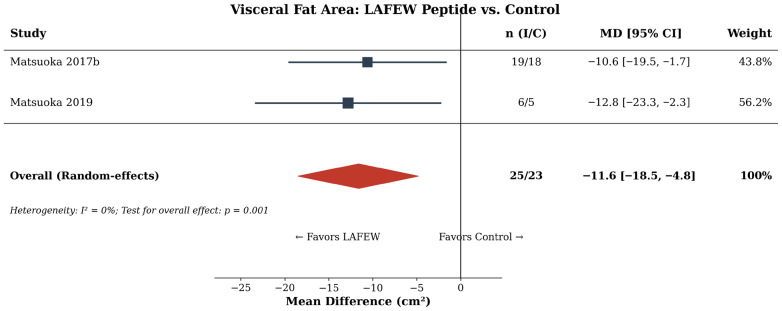
Forest plot of the effect of lactic-fermented egg white (LAFEW) peptide on visceral fat area [15,38]; squares represent individual study effect sizes, with square size proportional to study weight; and horizontal lines represent 95% confidence intervals. The diamond represents the pooled estimate, with diamond width indicating the 95% CI. The vertical solid line represents the null effect (MD = 0). n (I/C), number of participants in the intervention/control group; MD, mean difference; CI, confidence interval.

Two RCTs [16,38] evaluated the effect of LAFEW on VFA in overweight Japanese adults. The pooled MD was −11.6 cm^2^ (95% CI: −18.5 to −4.8; *I*^2^ = 0%), indicating a statistically significant reduction in VFA compared with whey protein control (Figure 3). Both trials used CT-measured VFA in participants with baseline VFA ≥ 100 cm^2^. The absence of heterogeneity (*I*^2^ = 0%) suggests consistency across studies, though both originated from the same Japanese research group with sponsorship from the Kewpie Corporation. The GRADE certainty was rated as very low, downgraded for risk of bias, imprecision, and indirectness (single research group, industry-sponsored).

### 3.8. SBP (NWT-03)

**Figure 4:**
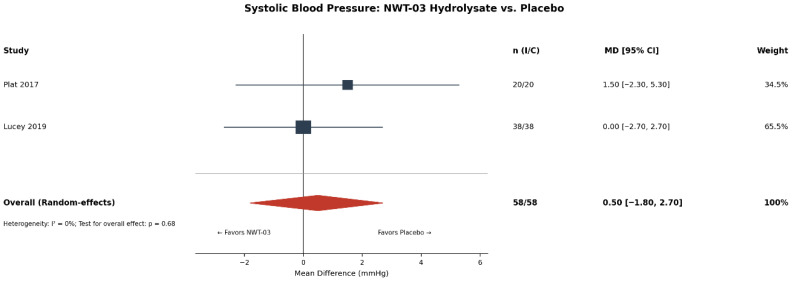
Forest plot of the effect of NWT-03 egg protein hydrolysate on systolic blood pressure [14,40]; Squares represent individual study effect sizes, with square size proportional to study weight; horizontal lines represent 95% confidence intervals. The diamond represents the pooled estimate, with diamond width indicating the 95% CI. The vertical solid line represents the null effect (MD = 0). n (I/C), number of participants in the intervention/control group; MD, mean difference; CI, confidence interval.

Two RCTs [14,40] evaluated the effect of NWT-03 egg protein hydrolysate on SBP. The pooled MD was +0.5 mmHg (95% CI: −1.8 to +2.7; *I*^2^ = 0%), indicating no statistically significant effect on BP (Figure 4). The null result reflects the conflicting findings between Plat et al.’s [14] dose-finding trial, which reported significant BP reductions at the 5 g dose in a subgroup with mild hypertension, and Lucey et al.’s [40] confirmatory trial in older adults with mildly elevated BP, which found no effect at a lower dose (3 g/day). GRADE certainty was not formally assessed for this null result.

Leave-one-out sensitivity analysis (Table S4, Figure S3), narrative synthesis of non-pooled outcomes, and publication bias (Figure S4) are described in Supplementary File S3.

## 4. Discussion

### 4.1. Principal Findings

This SR and meta-analysis is, to our knowledge, the first to comprehensively synthesize RCT evidence on egg-derived protein and peptide supplementation across all adult health outcomes. Thirty RCTs involving 1938 participants were included, encompassing five distinct categories of egg-derived interventions: intact egg white protein for dialysis patients, LAFEW peptides for metabolic health, NWT-03 egg protein hydrolysate for cardiovascular and cognitive outcomes, egg protein for exercise performance and sarcopenia, and other clinical applications including enteral nutrition in critically ill patients, nutritional supplementation in malnourished elderly patients, mental fatigue reduction, and stress-related cognitive outcomes.

Four outcomes were amenable to quantitative pooling. Egg white supplementation in dialysis patients was associated with a significant increase in serum albumin and decrease in serum phosphorus, though with substantial heterogeneity for albumin (*I*^2^ = 82.4%). LAFEW peptide supplementation reduced visceral fat, while NWT-03 hydrolysate showed no significant effect on systolic blood pressure. The overall certainty of evidence was very low for albumin and low for phosphorus by GRADE assessment.

### 4.2. Comparison with Previous Literature

The albumin finding is directionally consistent with the Cochrane review on oral protein supplements for dialysis patients [19], which found insufficient evidence to recommend any specific protein source. However, the clinical confidence in the present findings is weakened by three observations: the albumin result was driven predominantly by studies at high risk of bias, with the single remaining low-to-moderate risk study [8] showing a non-significant effect (MD: +0.20 g/dL); the heterogeneity was primarily attributable to one study [9]; and the practical significance of a 0.42 g/dL increase may vary depending on baseline nutritional status. Nevertheless, the consistent direction of effect across all four studies and the biologically plausible mechanism—the provision of high-quality protein with a favorable phosphorus-to-protein ratio—support the potential utility of egg white supplementation as a dietary adjunct in this population.

The phosphorus reduction is particularly clinically relevant, as hyperphosphatemia is a major contributor to cardiovascular mortality in dialysis patients, and egg whites represent a protein source with exceptionally low phosphorus content relative to other animal proteins.

The LAFEW trials demonstrated a significant reduction in visceral fat area, though both studies originated from a single Japanese research group with industry funding [15,16,38]. The cholesterol-lowering effect observed in one trial [15] was modest and has not been independently replicated. Independent replication in diverse populations is required before these findings can be considered generalizable.

The NWT-03 trials represent the most methodologically rigorous body of evidence in this review, with all seven publications rated as low risk of bias. Despite this methodological strength, the clinical findings are inconsistent: the BP meta-analysis showed no significant effect, while individual studies reported conflicting results on arterial stiffness and vascular outcomes [14,23,25,41]. The cognitive findings [24,36] are novel and intriguing but require replication, particularly given the exploratory nature of the endpoints and the modest effect sizes.

Regarding MPS, the narrative findings corroborate the broader literature on protein quality and postexercise anabolism. The dose–response curve for egg protein [42] aligns with the approximately 20 g threshold identified in a prior large-scale meta-regression [18]. The finding that thermal processing enhances egg protein digestibility and MPS [46] is consistent with earlier stable-isotope work [5], reinforcing that the consumption of raw eggs may actually impair rather than enhance postexercise protein utilization.

Two studies [47,52] converge on the concept that egg yolk components confer additional anabolic properties beyond the protein fraction alone: one demonstrated acutely enhanced myofibrillar MPS from whole eggs despite identical leucine availability [52], while the other showed chronically superior strength gains from whole eggs versus egg whites during 12 weeks of resistance training [47]. The dissociation between strength gains and hypertrophy in the latter study is notable and warrants further investigation into potential neuromuscular or hormonal mechanisms, including the observed testosterone elevation in the whole-egg group.

The Fortetropin trials represent an emerging line of investigation into egg yolk-derived bioactive compounds targeting the myostatin pathway. The progression from the initial positive findings in young resistance-trained males [53] to the proteome-wide demonstration of enhanced MPS in older adults [54] provides a mechanistic framework for Fortetropin’s anabolic effects. However, one trial [55] reported null findings on disuse atrophy prevention despite circulating myostatin suppression, highlighting the complexity of muscle wasting and the gap between circulating biomarker changes and clinically meaningful tissue-level outcomes. All three Fortetropin trials were industry-sponsored, and independent replication is needed before firm conclusions can be drawn.

The longer-term exercise studies [43,44,45] collectively suggest that egg white protein supplementation during resistance training does not consistently produce superior gains in muscle mass or strength compared with carbohydrate or control conditions. These findings are consistent with the broader protein supplementation literature, which suggests that protein timing and dose, rather than protein source per se, are the primary determinants of resistance training adaptations.

### 4.3. Dose–Duration–Effect Relationships

The heterogeneity of interventions across the included studies, spanning doses from 3 g/day (NWT-03) to 40 g/day (enteral egg white), durations from single acute doses to 36 weeks, and product types from intact protein to enzymatically hydrolyzed peptides, precludes definitive conclusions about optimal dosing. For egg white protein in dialysis patients, interventions providing approximately 24 g protein three times weekly for 8 weeks (Azmandian et al.) appeared effective, while shorter durations and lower doses have not been tested. For NWT-03, a dose–response pattern was suggested by Plat et al. 2017 [14], with 5 g/day showing greater effects than 1 g or 2 g, but this was not confirmed in the subsequent confirmatory trial by Lucey et al., using 3 g/day. For LAFEW, the 8 g/day dose consistently outperformed 4 g and 6 g doses across trials. These observations underscore the need for future trials to systematically evaluate dose–response relationships and the minimum effective durations for each egg-derived product category.

### 4.4. Cognitive Outcomes

Regarding cognitive outcomes, two NWT-03 trials reported promising but preliminary findings. Gravesteijn et al. demonstrated improved executive function (reaction time, *p* < 0.001) after 4 weeks of supplementation in adults with metabolic syndrome, while Adams et al. reported sex-specific improvements in multitasking and working memory after 36 weeks in older adults. Additionally, Markus et al. showed that a tryptophan-rich egg protein hydrolysate acutely improved vigilance and cognitive performance under stress conditions. These findings are notable given the mechanistic plausibility linking ACE-inhibitory peptides to cerebrovascular function and tryptophan availability to serotonergic neurotransmission. However, the cognitive evidence remains exploratory, with modest sample sizes (n = 35–76 per trial), heterogeneous outcome measures, and no independent replication. Larger, pre-registered trials with standardized cognitive batteries are needed before cognitive health claims for egg-derived peptides can be supported.

### 4.5. Clinical Implications

Importantly, the current evidence is derived exclusively from specific clinical populations—primarily maintenance dialysis patients with established hypoalbuminemia—and should not be extrapolated to support the use of oral albumin-containing functional foods or dietary supplements marketed to the general public. The modest improvements in serum albumin observed in this review reflect the replacement of high-phosphorus protein sources with egg white under clinical supervision, a context that differs fundamentally from over-the-counter supplements claiming to raise serum albumin in healthy individuals.

### 4.6. Future Perspectives

Several directions emerge from this synthesis. First, adequately powered, double-blind RCTs in dialysis patients are urgently needed, as the current albumin evidence is driven by high-risk studies and no blinded trial exists in this population. Second, independent replication of the LAFEW visceral fat findings in non-Japanese populations would strengthen the evidence base. Third, the cognitive effects of NWT-03 represent a novel and potentially high-impact area that warrants further investigation with larger samples and longer durations. Fourth, the Fortetropin research trajectory—particularly the disconnect between myostatin suppression and functional outcomes—requires mechanistic studies to identify whether the anabolic effects are clinically translatable. Finally, future research should investigate whether longer-term NWT-03 supplementation produces sustained improvements in vascular and cognitive function, and whether the sex-specific cognitive effects reported in one trial [36] are reproducible. Notably, no RCTs have examined egg-derived supplementation in malnutrition associated with inflammatory bowel disease or cancer, representing important populations for future investigation.

### 4.7. Strengths and Limitations

This study has several methodological strengths. It is the first SR to comprehensively synthesize all RCT evidence on egg-derived protein and peptide supplementation across all adult health outcomes. The search strategy was broad and inclusive, covering four major databases plus trial registries. The identification of companion reports from shared trial registrations (NCT02561663, NCT04831203) prevented potential double-counting. Risk of bias assessment using the Cochrane RoB 2 tool and evidence grading using the GRADE framework provided transparent quality evaluation.

However, several limitations should be acknowledged. First, the small number of studies per meta-analyzed outcome (2–4 RCTs) limited statistical power for heterogeneity assessment, subgroup analysis, and publication bias testing. Second, the preponderance of open-label designs in dialysis trials introduces potential performance and detection bias that cannot be adequately addressed through sensitivity analysis. Third, the geographic concentration of certain trial categories (LAFEW trials exclusively in Japan, NWT-03 trials predominantly in The Netherlands) limits the generalizability of findings across populations with different dietary patterns and genetic backgrounds. Fourth, 4 of the 30 included studies had industry sponsorship (three Fortetropin trials by MYOS Corporation, LAFEW trials by Kewpie Corporation), which may introduce reporting bias. Fifth, the heterogeneity of egg-derived interventions—spanning intact egg white, lactic-fermented peptides, ovalbumin hydrolysates, Fortetropin, and tryptophan-rich hydrolysates—precluded comprehensive pooling and limited the ability to draw unified conclusions about “egg protein” as a class.

## 5. Conclusions

Current evidence for egg-derived protein supplementation is limited to specific clinical populations—primarily maintenance dialysis patients with hypoalbuminemia—and should not be extrapolated to justify oral albumin-containing functional foods marketed to the general public. The modest benefits observed in this review were achieved through dietary egg white substitution under clinical supervision, which differs fundamentally from commercial supplements claiming to raise serum albumin in healthy individuals. Rigorous, population-specific trials are essential before any clinical recommendations can be made.

## Figures and Tables

**Table 1:** Detailed study characteristics by intervention type.

Author	Ref	Year	Country	Design	N	Population	Intervention	Comparator	Duration	Primary Outcome	Key Results
Category 1: Intact Egg White Protein—Dialysis/CKD (n = 5)											
González-Espinoza	[7]	2005	Mexico	RCT, open-label, parallel	28	CAPD patients with malnutrition	Egg albumin-based supplement + dietary counseling	Dietary counseling alone	6 mo	Serum albumin, nPNA, SGA	Albumin ↑ 2.64 → 3.05 g/dL (*p* < 0.05); malnutrition ↓ 28% vs. 6%
Guida	[8]	2019	Italy	RCT	23	HD, hyperphosphatemia	Egg white 2×/week	Standard diet	3 mo	Serum P	Effective P control
Azmandian	[9]	2022	Iran	Open-label controlled trial	150 (128 analyzed)	HD, serum P ≥ 5.5 mg/dL	Six egg whites (24 g protein) 3×/week	Control	8 wk	Serum P, albumin	P ↓ 4.5 vs. 6.7 mg/dL (*p* = 0.001); albumin ↑; cholesterol ↓
Javadian	[10]	2024	Iran	RCT, 4-arm parallel	98	Maintenance HD patients	24 g egg white ± 600 mg niacin	Control (routine care)	8 wk	Serum P, albumin, Kt/V	Albumin ↑; Kt/V and URR ↑ in EW group
Azmandian	[37]	2025	Iran	Open-label parallel	107	HD, Hb < 12 g/dL	Egg white pack (24 g protein) 3×/week	Control	8 wk	Hemoglobin, serum iron, ferritin, iron/TIBC, albumin, EPO dose	Hb significantly ↑ in EW group
Category 2: Egg-Derived Protein—Metabolic/Weight Management (n = 4)											
Matsuoka (a)	[15]	2017	Japan	RCT, DB, 3-arm parallel	88	Mild hypercholesterolemia, men	LAFEW 4 g, 6 g, or 8 g protein/day	Dose comparison	8 wk	Serum TC, LDL-C	8 g: TC ↓ 11.0 ± 3.7 mg/dL (*p* < 0.05 vs. 4 g)
Matsuoka (b)	[16]	2017	Japan	RCT, DB, parallel	37	VFA ≥ 100 cm^2^, age ≥ 40	LAFEW 8 g protein/day	Whey protein 8 g/day	12 wk	Visceral fat area	VFA ↓ 8.89 cm^2^ vs. +1.71 cm^2^ (*p* < 0.05)
Matsuoka	[38]	2019	Japan	RCT, DB, 3-arm pilot	22	Males, BMI ≥ 24, waist ≥ 85 cm	LAFEW 6 g or 8 g protein/day	Whey protein 8 g/day	8 wk	VFA, body weight	8 g LAFEW: VFA ↓ 13.2 ± 4.7 cm^2^
Brun	[39]	2018	France	RCT + follow-up	22 + 337	Obese, low protein intake	Ovamine^®^ 1.2–1.4 g/kg/d protein	Low-fat high-protein diet	2 + 18 mo	Body weight, fat mass	Weight ↓ 1.97 kg, fat ↓ 3.2 kg (2 mo); sustained at 18 mo
Category 3: NWT-03 Egg Protein Hydrolysate (n = 7 publications from 5 unique trials)											
Plat	[14]	2017	The Netherlands	RCT, crossover, DB, dose-finding	~92	Normotensive to mild HTN	NWT-03 1 g, 2 g, or 5 g/day	Placebo	4 wk/period	24 h ambulatory BP	2 g: BP ↓ in mild HTN; 5 g: night SBP ↓ 14.8, DBP ↓ 8.4 mmHg
Lucey	[40]	2019	Ireland	RCT, crossover, DB	75 (65)	Adults 50–70 y, SBP 130–150	Ovalbumin hydrolysate 3 g/day	Placebo 3 g/day	6 wk × 2	Office/central BP, cfPWV	NEGATIVE: No effect on BP, arterial stiffness, lipids
Plat	[41]	2019	The Netherlands	RCT, crossover, DB	40	Overweight/obese, IGT/T2DM, ~60 y	NWT-03 5 g/day	Maltodextrin	3 days (acute)	cr-PWV, glucose, lipids	PWV ↓; glucose ↓; insulin ↓; HDL ↑; TG ↓
Nijssen	[23]	2023	The Netherlands	RCT, crossover, DB	76	Metabolic syndrome	NWT-03 5 g/day	Placebo	27 days/period	cfPWV, PWVc-r, CAIxHR75, pulse pressure, fasting lipids, glucose, insulin	No effect on arterial stiffness; modest ↓ fasting pulse pressure
Gravesteijn	[24]	2023	The Netherlands	RCT, crossover, DB	76	Metabolic syndrome, 60 ± 10 y	NWT-03 5 g/day	Maltodextrin 5 g/day	4 wk/period	Cognitive function, BDNF	Improved executive function (reaction time *p* < 0.001); no effect on BDNF
Adams (endothelial)	[25]	2025	The Netherlands	RCT, parallel, DB	44 (40)	Older adults 60–75 y, overweight/obese	NWT-03 5.7 g/day	Maltodextrin	36 wk	FMD, arterial stiffness	FMD improved (endothelial function); no effect on PWV
Adams (cognitive)	[36]	2024	The Netherlands	RCT, parallel, DB	44	Older adults, elevated cognitive failures	NWT-03 5.7 g/day	Maltodextrin	36 wk	Cognitive (CANTAB), CBF	Women: improved multitasking, working memory
Category 4: Egg Protein—Exercise, Muscle Protein Synthesis, and Sarcopenia (n = 10)											
Moore	[42]	2009	Canada	RCT, crossover (5 conditions)	6	Resistance-trained young men	0, 5, 10, 20, or 40 g egg protein post-exercise	Dose comparison	Acute (5 h)	Muscle protein synthesis	MPS maximally stimulated at 20 g; leucine oxidation ↑ > 20 g
Van Vliet	[52]	2017	USA	RCT, crossover	10	Resistance-trained men, 21 ± 1 y	Whole eggs (18 g protein, 17 g fat)	Egg whites (18 g protein, 0 g fat)	Acute (5 h)	Myofibrillar protein synthesis	Whole eggs 40% greater MPS than egg whites (*p* = 0.04)
Fuchs	[46]	2022	The Netherlands	RCT, parallel (3 arms)	45	Healthy young men, 24 y	5 raw eggs or 5 boiled eggs (~30 g protein)	Control breakfast (~5 g protein)	Acute (6 h)	Myofibrillar protein synthesis	Both egg groups MPS ↑ 2–4 fold; no diff raw vs. boiled
Hida	[43]	2012	Japan	RCT, DB, parallel	30	Female athletes, 18–22 y	Egg white 15 g/day pre-training	Maltodextrin 17.5 g/day	8 wk	FFM, 1RM strength	No group diff in FFM or 1RM; serum urea/citrulline ↑ in EW
Kato	[44]	2011	Japan	RCT	30	Healthy young adults	15 g EWP + light resistance exercise	Control	5 wk	Muscle mass, strength	↑ muscle CSA, ↑ strength, ↓ fat mass
Ullevig	[45]	2022	USA	RCT, DB, pilot	29	Latina women ≥ 60 y, reduced strength	Dried egg white 20 g/day	Maltodextrin (isocaloric)	6 mo	ASMM, handgrip, arm curls	No between-group diff; within EW: handgrip ↑, arm curls ↑
Bagheri	[47]	2021	Iran	RCT, parallel	30	Resistance-trained males, ~24 y	6 egg whites post-exercise	3 whole eggs	12 wk	Muscle CSA, strength	No diff in mass; whole egg > EW for testosterone, strength
Sharp	[53]	2016	USA	RCT, DB, parallel	37	Resistance-trained college males	Fortetropin 6.6 g or 19.8 g/day	Macronutrient-matched placebo	12 wk	Lean mass, strength, myostatin	Lean mass + 1.7 kg; myostatin ↓ 18–22%
Evans	[54]	2021	USA	RCT, DB, parallel	20	Older adults, 66 ± 5 y	Fortetropin 19.8 g/day	Cheese powder placebo	21 days	Muscle protein FSR	MPS ↑ 18% vs. placebo
Lim	[55]	2023	Canada	RCT, DB, parallel	24	Healthy young men, 22 ± 2 y	Fortetropin 19.8 g/day during immobilization	Cheese powder placebo	6 wk	Muscle CSA, myostatin	Prevented myostatin rise; no protection against atrophy
Category 5: Miscellaneous Populations and Outcomes (n = 4)											
Bhurayanontachai	[48]	2016	Thailand	RCT, DB, non-inferiority	40	Acute respiratory failure, ICU	Egg white 40 g/day + EN	Casein 40 g/day + EN	7 days	Serum prealbumin, CRP	EW non-inferior to casein for prealbumin
Mukmin	[49]	2024	Indonesia	RCT, placebo-controlled	46	Malnourished elderly inpatients	Egg white extract 30 g/day	Placebo	2 wk	Serum albumin, IGF-1	Albumin 2.80 → 3.70 g/dL; IGF-1 1.74 → 24.74 ng/mL
Oe	[50]	2020	Japan	RCT, DB × 2 studies	19 + 74	Japanese adults, self-perceived fatigue	Peptifine (EWH) 5 g/day	Placebo	2 wk	Mental fatigue, antioxidant	Mental fatigue ↓ vs. placebo; antioxidant capacity ↑
Markus	[51]	2010	The Netherlands	RCT, crossover, DB	35	Healthy adults, stress-stratified	Tryptophan-rich egg hydrolysate	Placebo	Acute	Plasma TRP/LNAA, mood, vigilance	↑ TRP ratio, ↑ vigilance, ↑ performance

Abbreviations: ASMM, appendicular skeletal muscle mass; BDNF, brain-derived neurotrophic factor; BP, blood pressure; CAIxHR75, central augmentation index corrected for heart rate at 75 bpm; CANTAB, Cambridge Neuropsychological Test Automated Battery; CAPD, continuous ambulatory peritoneal dialysis; CBF, cerebral blood flow; cfPWV, carotid–femoral pulse wave velocity; CRP, *C*-reactive protein; CSA, cross-sectional area; DB, double-blind; DBP, diastolic blood pressure; EN, enteral nutrition; EPO, erythropoietin; EW, egg white; EWH, egg white hydrolysate; EWP, egg white protein; FFM, fat-free mass; FMD, flow-mediated dilation; FSR, fractional synthetic rate; HD, hemodialysis; HTN, hypertension; IGF-1, insulin-like growth factor 1; IGT, impaired glucose tolerance; LAFEW, lactic-acid-fermented egg white; LDL-C, low-density lipoprotein cholesterol; MPS, muscle protein synthesis; nPNA, normalized protein nitrogen appearance; PWVc-r, carotid-to-radial pulse wave velocity; PWV, pulse wave velocity; RCT, randomized controlled trial; SBP, systolic blood pressure; SGA, Subjective Global Assessment; T2DM, type 2 diabetes mellitus; TC, total cholesterol; TG, triglycerides; TIBC, total iron-binding capacity; TRP/LNAA, tryptophan to large neutral amino acids ratio; URR, urea reduction ratio; VFA, visceral fat area; 1RM, one-repetition maximum.

## Data Availability

The original contributions presented in this study are included in the article/Supplementary Materials. Further inquiries can be directed to the corresponding authors.

## Supplementary Materials

The following supporting information can be downloaded at: https://www.mdpi.com/article/10.3390/nu18071054/s1, File S1: PRISMA 2020 guidelines; File S2: Search Strategy for Each Database; File S3: Methods; Figure S1: PRISMA 2020 flow diagram of study selection; Figure S2: Risk of bias assessment of included studies; Figure S3: Leave-one-out sensitivity analysis for the serum albumin meta-analysis; Figure S4: Funnel plot for the publication bias assessment; Table S1: Existing Systematic Reviews Related to Egg Protein Supplementation; Table S2: Risk of Bias 2 (RoB 2) Assessment of Included Studies; Table S3: Summary of Meta-Analysis Results; Table S4: Leave-One-Out Sensitivity Analysis—Serum Albumin.
