# Supplementary material for: Oral Egg-Derived Protein and Peptide Supplementation for Health Outcomes in Adults: Systematic Review and Meta-Analysis
Source: Nutrients. 2026 Mar 26;18(7):1054. doi: 10.3390/nu18071054 (PMC13074808; doi:10.3390/nu18071054)
Supplement: Supplementary file 1 [file nutrients-18-01054-s001.zip › nutrients-4196461-supplementary.pdf]

Supplementary file S1

Section and Topic	Item #	Checklist item	Location where item is reported
**TITLE**			
Title	1	Identify the report as a systematic review.	p.1
**ABSTRACT**			
Abstract	2	See the PRISMA 2020 for Abstracts checklist.	pp.4-5
**INTRODUCTION**			
Rationale	3	Describe the rationale for the review in the context of existing knowledge.	pp.5-6
Objectives	4	Provide an explicit statement of the objective(s) or question(s) the review addresses.	pp.6-7
**METHODS**			
Eligibility criteria	5	Specify the inclusion and exclusion criteria for the review and how studies were grouped for the syntheses.	p.7; Supplementary file 2
Information sources	6	Specify all databases, registers, websites, organisations, reference lists and other sources searched or consulted to identify studies. Specify the date when each source was last searched or consulted.	p.4; Supplementary file 2
Search strategy	7	Present the full search strategies for all databases, registers and websites, including any filters and limits used.	Supplementary file 3
Selection process	8	Specify the methods used to decide whether a study met the inclusion criteria of the review, including how many reviewers screened each record and each report retrieved, whether they worked independently, and if applicable, details of automation tools used in the process.	Supplementary file 2
Data collection process	9	Specify the methods used to collect data from reports, including how many reviewers collected data from each report, whether they worked independently, any processes for obtaining or confirming data from study investigators, and if applicable, details of automation tools used in the process.	Supplementary file 2
Data items	10a	List and define all outcomes for which data were sought. Specify whether all results that were compatible with each outcome domain in each study were sought (e.g. for all measures, time points, analyses), and if not, the methods used to decide which results to collect.	Supplementary file 2
	10b	List and define all other variables for which data were sought (e.g. participant and intervention characteristics, funding sources). Describe any assumptions made about any missing or unclear information.	Supplementary file 2
Study risk of bias assessment	11	Specify the methods used to assess risk of bias in the included studies, including details of the tool(s) used, how many reviewers assessed each study and whether they worked independently, and if applicable, details of automation tools used in the process.	Supplementary file 2
Effect measures	12	Specify for each outcome the effect measure(s) (e.g. risk ratio, mean difference) used in the synthesis or presentation of results.	Supplementary file 2
Synthesis methods	13a	Describe the processes used to decide which studies were eligible for each synthesis (e.g. tabulating the study intervention characteristics and comparing against the planned groups for each synthesis (item #5)).	Supplementary file 2
	13b	Describe any methods required to prepare the data for presentation or synthesis, such as handling of missing summary statistics, or data conversions.	Supplementary file 2
	13c	Describe any methods used to tabulate or visually display results of individual studies and syntheses.	Supplementary file 2
	13d	Describe any methods used to synthesize results and provide a rationale for the choice(s). If meta-analysis was performed, describe the	Supplementary file 2

Section and Topic	Item #	Checklist item	Location where item is reported
		model(s), method(s) to identify the presence and extent of statistical heterogeneity, and software package(s) used.	
	13e	Describe any methods used to explore possible causes of heterogeneity among study results (e.g. subgroup analysis, meta-regression).	Supplementary file 2
	13f	Describe any sensitivity analyses conducted to assess robustness of the synthesized results.	Supplementary file 2
Reporting bias assessment	14	Describe any methods used to assess risk of bias due to missing results in a synthesis (arising from reporting biases).	Supplementary file 2
Certainty assessment	15	Describe any methods used to assess certainty (or confidence) in the body of evidence for an outcome.	Supplementary file 2
**RESULTS**			
Study selection	16a	Describe the results of the search and selection process, from the number of records identified in the search to the number of studies included in the review, ideally using a flow diagram.	pp.7-8; Figure 1
	16b	Cite studies that might appear to meet the inclusion criteria, but which were excluded, and explain why they were excluded.	p.8
Study characteristics	17	Cite each included study and present its characteristics.	pp.8-10; Table 1 (p.26)
Risk of bias in studies	18	Present assessments of risk of bias for each included study.	pp.10-11; Figure 2 (p.24)
Results of individual studies	19	For all outcomes, present, for each study: (a) summary statistics for each group (where appropriate) and (b) an effect estimate and its precision (e.g. confidence/credible interval), ideally using structured tables or plots.	pp.11-12; Figures 3-6 (pp.24-25)
Results of syntheses	20a	For each synthesis, briefly summarise the characteristics and risk of bias among contributing studies.	pp.11-12
	20b	Present results of all statistical syntheses conducted. If meta-analysis was done, present for each the summary estimate and its precision (e.g. confidence/credible interval) and measures of statistical heterogeneity. If comparing groups, describe the direction of the effect.	pp.11-12; Table S3
	20c	Present results of all investigations of possible causes of heterogeneity among study results.	p.12
	20d	Present results of all sensitivity analyses conducted to assess the robustness of the synthesized results.	p.12; Table S4
Reporting biases	21	Present assessments of risk of bias due to missing results (arising from reporting biases) for each synthesis assessed.	p.12
Certainty of evidence	22	Present assessments of certainty (or confidence) in the body of evidence for each outcome assessed.	pp.4, 12
**DISCUSSION**			
Discussion	23a	Provide a general interpretation of the results in the context of other evidence.	pp.13-15
	23b	Discuss any limitations of the evidence included in the review.	pp.16-17
	23c	Discuss any limitations of the review processes used.	pp.16-17
	23d	Discuss implications of the results for practice, policy, and future research.	p.16
**OTHER INFORMATION**			

Section and Topic	Item #	Checklist item	Location where item is reported
Registration and protocol	24a	Provide registration information for the review, including register name and registration number, or state that the review was not registered.	p.3 (PROSPERO: CRD420261295226)
	24b	Indicate where the review protocol can be accessed, or state that a protocol was not prepared.	Supplementary file 2
	24c	Describe and explain any amendments to information provided at registration or in the protocol.	N/A
Support	25	Describe sources of financial or non-financial support for the review, and the role of the funders or sponsors in the review.	p.2
Competing interests	26	Declare any competing interests of review authors.	p.1
Availability of data, code and other materials	27	Report which of the following are publicly available and where they can be found: template data collection forms; data extracted from included studies; data used for all analyses; analytic code; any other materials used in the review.	p.3

From: Page MJ, McKenzie JE, Bossuyt PM, Boutron I, Hoffmann TC, Mulrow CD, et al. The PRISMA 2020 statement: an updated guideline for reporting systematic reviews. BMJ 2021;372:n71. doi: 10.1136/bmj.n71. This work is licensed under CC BY 4.0. To view a copy of this license, visit https://creativecommons.org/licenses/by/4.0/

Supplementary file S2:

Supplementary file S2

Search Strategy for Each Database

Oral Egg-Derived Protein and Peptide Supplementation for Health Outcomes in Adults:
A Systematic Review and Meta-Analysis of Randomized Controlled Trials

Search date: January 31, 2026

Language restrictions: None

Date restrictions: Inception to January 31, 2026

1. PubMed/MEDLINE

Concept 1: Egg-derived protein/peptide intervention

#1 `"Egg Proteins"[MeSH Terms]: 34103`

#2 `"Egg White"[MeSH Terms]: 3280`

#3 `"Egg Proteins, Dietary"[MeSH Terms]: 25795`

#4 `"Ovalbumin"[MeSH Terms]: 24024`

#5 `"egg white"[Title/Abstract]: 7691`

#6 `"egg protein*"[Title/Abstract]: 1028`

#7 `"egg albumin"[Title/Abstract]: 1306`

#8 `"ovalbumin"[Title/Abstract]: 22763`

#9 `"egg hydrolysate*"[Title/Abstract]: 12`

#10 `"egg peptide*"[Title/Abstract]: 37`

#11 `"egg-derived"[Title/Abstract]: 328`

#12 `"NWT-03"[Title/Abstract]: 6`

#13 "lactic-fermented egg white"[Title/Abstract]: 5

#14 "LAFEW"[Title/Abstract]: 1

#15 "ovokinin"[Title/Abstract]: 21

#16 "IRW"[Title/Abstract] AND (egg[Title/Abstract] OR peptide[Title/Abstract]): 35

#17 "egg white protein*"[Title/Abstract]: 907

#18 "egg protein hydrolysate*"[Title/Abstract]: 21

#19 #1 OR #2 OR #3 OR #4 OR #5 OR #6 OR #7 OR #8 OR #9 OR #10 OR #11 OR #12 OR #13 OR #14 OR #15 OR #16 OR #17 OR #18: 52111

Concept 2: Randomized controlled trial (study design filter)

#20 "Randomized Controlled Trial"[Publication Type]: 654856

#21 "Controlled Clinical Trial"[Publication Type]: 745701

#22 "Randomized Controlled Trials as Topic"[MeSH Terms]: 196996

#23 "Random Allocation"[MeSH Terms]: 109379

#24 "Double-Blind Method"[MeSH Terms]: 187943

#25 "Single-Blind Method"[MeSH Terms]: 36137

#26 "Clinical Trials as Topic"[MeSH Terms]: 421870

#27 "randomized"[Title/Abstract]: 803946

#28 "randomised"[Title/Abstract]: 156042

#29 "placebo"[Title/Abstract]: 274391

#30 "randomly"[Title/Abstract]: 481333

#31 "trial"[Title/Abstract]: 940904

#32 "groups"[Title/Abstract]: 3033546

#33 #20 OR #21 OR #22 OR #23 OR #24 OR #25 OR #26 OR #27 OR #28 OR #29 OR #30 OR #31 OR #32: 4651852

Combined search

#34 #19 AND #33: 6013

No language or date filters applied.

2. Embase (via Ovid)

Concept 1: Egg-derived protein/peptide intervention

```
#1  exp egg protein/ : 2862
#2  exp egg white/ : 5532
#3  exp ovalbumin/ : 30630
#4  exp egg albumin/ : 30630
#5  (egg white or egg protein* or egg albumin or ovalbumin).ti,ab,kw. : 37726
#6  (egg hydrolysate* or egg peptide* or egg-derived).ti,ab,kw. : 462
#7  (NWT-03 or NWT03).ti,ab,kw. : 10
#8  (lactic-fermented egg white or LAFEW).ti,ab,kw. : 6
#9  (ovokinin or IRW peptide or IRW tripeptide).ti,ab,kw. : 23
#10  (egg white protein* or egg protein hydrolysate*).ti,ab,kw. : 925
#11  1 or 2 or 3 or 4 or 5 or 6 or 7 or 8 or 9 or 10 : 50412
```

Concept 2: Randomized controlled trial (study design filter)

```
#12  exp randomized controlled trial/ : 1145948
#13  exp controlled clinical trial/ : 1337137
#14  exp crossover procedure/ : 107499
#15  exp double blind procedure/ : 317756
#16  exp single blind procedure/ : 85528
#17  (randomized or randomised or placebo or randomly or trial or RCT).ti,ab,kw. : 2910475
#18  (crossover or cross-over or "cross over").ti,ab,kw.: 160194
#19  ((singl* or doubl* or trebl* or tripl*) adj3 (blind* or mask*)).ti,ab,kw.: 393120
#20  12 or 13 or 14 or 15 or 16 or 17 or 18 or 19: 4395867
```

Exclusion filter

#21 (animal/ or nonhuman/) not human/ : 7162019

Combined search

#22 11 and 20: 3123

#23 22 not 21: 1669

No language or date limits applied. Deduplicated against PubMed results.

3. Cochrane Central Register of Controlled Trials (CENTRAL)

Concept 1: Egg-derived protein/peptide intervention

#1 [mh "Egg Proteins"]: 114

#2 [mh "Egg White"]: 46

#3 [mh "Ovalbumin"]: 42

#4 ("egg white" OR "egg protein" OR "egg albumin" OR ovalbumin):ti,ab,kw: 500

#5 ("egg hydrolysate" OR "egg peptide" OR "egg-derived"):ti,ab,kw: 64

#6 ("NWT-03" OR "NWT03"):ti,ab,kw: 11

#7 ("lactic-fermented egg white" OR "LAFEW"):ti,ab,kw: 5

#8 (ovokinin OR "IRW peptide" OR "IRW tripeptide"):ti,ab,kw: 0

#9 ("egg white protein" OR "egg protein hydrolysate"):ti,ab,kw: 54

#10 #1 OR #2 OR #3 OR #4 OR #5 OR #6 OR #7 OR #8 OR #9: 598

CENTRAL contains only clinical trials; no additional study design filter required.

No language or date limits applied.

4. Web of Science Core Collection

Concept 1: Egg-derived protein/peptide intervention

#1 TS=("egg white" OR "egg protein*" OR "egg albumin" OR "ovalbumin"): 17171

#2 TS=("egg hydrolysate*" OR "egg peptide*" OR "egg-derived"): #1 OR #2: 17369

#3 TS=("NWT-03" OR "NWT03" OR "lactic-fermented egg white" OR "LAFEW"): #1 OR # OR #3: 17369

#4 TS=("ovokinin" OR "IRW peptide" OR "IRW tripeptide"): #1 OR # OR #3 OR #4: 17371

#5 TS=("egg white protein*" OR "egg protein hydrolysate*"): #1 OR # OR #3 OR #4 OR #5: 17371

#6 #1 OR #2 OR #3 OR #4 OR #5: 17371

Concept 2: Randomized controlled trial (study design filter)

#7 TS=("randomized controlled trial" OR "randomised controlled trial" OR "RCT"): 196982

#8 TS=("randomized" OR "randomised" OR "placebo" OR "randomly"): #7 OR #8: 980079

#9 TS=("clinical trial" OR "controlled trial" OR "crossover" OR "cross-over"): #7 OR #8 OR 9: 1123850

#10 TS=("double-blind" OR "single-blind" OR "double blind" OR "single blind"): #7 OR #8 OR #9 OR 10: 1185055

#11 #7 OR #8 OR #9 OR #10: 1185055

Combined search

#12 #6 AND #11: 753

Indexes: SCI-EXPANDED, SSCI, ESCI. No language or date limits applied.

5. Additional Searches

5.1 Trial Registries

ClinicalTrials.gov (https://clinicaltrials.gov): Searched using "egg white" OR "egg protein" OR "egg albumin" OR "ovalbumin" OR "NWT-03" OR "LAFEW" in the intervention/treatment field. Filter: Interventional studies only. : 81

WHO ICTRP (https://trialsearch.who.int): Searched using "egg white" OR "egg protein" OR "egg albumin" OR "ovalbumin" OR "NWT-03" OR "LAFEW" in the intervention field.: 104

5.2 Reference List Screening

The reference lists of all included studies and relevant systematic reviews were manually screened to identify potentially eligible studies not captured by the electronic search. Reviews screened included:

• Kolahdouz-Mohammadi et al. (2020) — egg consumption and blood pressure

• Morton et al. (2018) — protein supplementation and resistance training

• Mah et al. (2020) — oral protein supplements in CKD/dialysis (Cochrane)

• Pripp (2008) — food-derived peptides and blood pressure

• Turpeinen et al. (2013) — bioactive tripeptides and blood pressure

• Liao et al. (2021) — antihypertensive peptides

Supplementary file S3

Methods

Protocol Registration and Reporting Guidelines

This SR and meta-analysis was conducted in accordance with the Preferred Reporting Items for Systematic Reviews and Meta-Analyses (PRISMA) 2020 statement (**Supplementary file S1**).[26] The protocol was registered prospectively in the International Prospective Register of Systematic Reviews (PROSPERO; CRD420261295226).

Information Sources and Search Strategy

A systematic literature search was performed in PubMed/MEDLINE, Embase-OVID, Cochrane Central Register of Controlled Trials (CENTRAL), and Web of Science from inception through January 31, 2026. The search strategy combined Medical Subject Headings (MeSH) terms and free-text keywords related to two key concepts: (1) egg-derived protein or peptide interventions (e.g., "egg white," "egg protein," "egg albumin," "ovalbumin," "egg hydrolysate," "egg peptide," "NWT-03," "lactic-fermented egg white," "LAFEW," "ovokinin," "IRW peptide") and (2) study design (e.g., "randomized controlled trial," "RCT," "clinical trial"). Boolean operators (AND, OR) were used to combine search terms within and between concepts. No language or date restrictions were applied. The detailed search strategy for each database is provided in **Supplementary file S2**.

In addition to electronic database searches, the reference lists of all included studies and relevant systematic reviews were manually screened to identify potentially eligible studies not captured by the electronic search. Trial registries, including ClinicalTrials.gov and the International Clinical Trials Registry Platform (ICTRP), were searched for completed but unpublished trials.

Eligibility Criteria

Studies were included if they met the following criteria based on the PICOS framework:

Population: Adults (≥18 years) of any health status, including but not limited to maintenance dialysis patients, elderly individuals with or without sarcopenia, athletes, and adults with metabolic syndrome, mild hypertension, or hypercholesterolemia.

Intervention: Oral supplementation with egg-derived protein or peptide preparations, including intact egg white protein (powder, liquid, or cooked egg white), egg protein hydrolysates (e.g., NWT-03, egg ovalbumin hydrolysate), LAFEW, egg white extract, or specific egg-derived bioactive peptides. Interventions providing egg protein as part of a multi-component supplement were included only if egg protein was the primary or sole protein source.

Comparator: Placebo (e.g., maltodextrin, whey protein, casein), no treatment, or usual care/diet.

Outcomes: Primary outcomes were serum albumin, serum phosphorus, systolic BP (SBP), diastolic BP (DBP), and body composition parameters (visceral fat area [VFA], lean body mass, fat mass). Secondary outcomes included serum cholesterol (total, LDL, HDL), muscle protein synthesis (MPS) rate, muscle strength, cognitive function, serum amino acid profiles, antioxidant capacity, and any reported adverse events.

Study design: RCTs of parallel-group or crossover design.

Studies were excluded if they: (1) used whole egg consumption (i.e., yolk and white combined) without isolating the egg white or egg-derived protein/peptide component; (2) administered egg protein parenterally or enterally via nasogastric tube (except in critically ill patients receiving enteral nutrition with egg white as a specified protein additive); (3) were non-randomized, observational, or single-arm studies; (4) included pediatric populations exclusively; or (5) reported only in vitro or animal model data

without a human clinical trial component.

Data Extraction

Data were extracted independently by two reviewers (CSB & EJG) using a standardized, piloted data extraction form. The following information was collected from each included study: first author, publication year, country, study design (parallel/crossover), sample size (randomized and analyzed), participant characteristics (age, sex, health condition, baseline serum albumin for dialysis studies), intervention details (type of egg protein/peptide product, dose, frequency, duration), comparator details, outcome measures with mean values and standard deviations (or standard errors, 95% confidence intervals [CIs], or medians with interquartile ranges) at baseline and endpoint or as change scores, and funding sources. For crossover trials, data from the first period were preferentially used when period-level data were available to avoid carryover effects; otherwise, crossover-specific data were extracted following methods recommended by Elbourne et al.[27]

When outcome data were reported only in figures, numerical values were extracted using validated digital graph-reading software (WebPlotDigitizer, version 4.6). Corresponding authors were contacted for missing or ambiguous data when necessary. For studies reporting outcomes at multiple time points, the final assessment time point was used for the primary analysis.

Risk of Bias Assessment

The risk of bias of each included RCT was assessed independently by two reviewers (CSB & EJG) using the Cochrane Risk of Bias 2 (RoB 2) tool.[28] For parallel-group trials, the standard RoB 2 tool was applied; for crossover trials, the adapted version for crossover designs was used. Risk of bias was evaluated across five domains: (D1) bias arising from the randomization process; (D2) bias due to deviations from intended interventions (assessed using the “effect of assignment” framework for intention-to-

treat analyses and the "effect of adhering" framework for per-protocol analyses); (D3) bias due to missing outcome data; (D4) bias in measurement of the outcome; and (D5) bias in selection of the reported result. Each domain was judged as "low risk," "some concerns," or "high risk," and an overall risk-of-bias judgment was derived according to the algorithm specified in the RoB 2 guidance document. Disagreements were resolved by consensus.

Statistical Analysis

Meta-analyses were performed when at least two studies reported comparable outcomes using similar interventions in similar populations. The mean difference (MD) with 95% CI was calculated as the summary effect measure for continuous outcomes. Random-effects models using the DerSimonian–Laird estimator were used for all analyses to account for anticipated between-study heterogeneity.[29]

Statistical heterogeneity was assessed using the Cochran Q test (significance threshold $p < 0.10$) and quantified with the I^2 statistic, with values of 25%, 50%, and 75% representing low, moderate, and high heterogeneity, respectively.[30] The between-study variance (τ^2) was reported for all pooled analyses. When I^2 exceeded 50%, prediction intervals (PIs) were calculated to estimate the range of true effects across settings.[31]

Pre-specified sensitivity analyses included: (1) leave-one-out analysis, removing each study iteratively to assess the influence of individual studies on the pooled estimate; (2) exclusion of studies rated as high risk of bias to evaluate the robustness of results to methodological quality; and (3) comparison of fixed-effect (inverse-variance) and random-effects models to assess the impact of the between-study variance assumption on the pooled effect.

Subgroup analyses were planned a priori by intervention duration (≤8 weeks vs. >8 weeks), intervention type (intact egg white protein vs. egg protein hydrolysate vs. lactic-fermented egg white), and participant health status (dialysis patients vs. non-dialysis populations). Interaction tests based on the Q statistic for subgroup differences were conducted, and subgroup analyses were interpreted cautiously given the limited number of studies per subgroup.

Exploratory meta-regression analyses were planned to examine associations between pre-specified covariates (baseline albumin level, intervention duration, sample size) and the effect size. However, given the small number of studies per outcome ($k < 10$ for all meta-analyzed outcomes), formal meta-regression was considered unreliable and results are reported for exploratory purposes only.[32]

Assessment of publication bias through visual inspection of funnel plots and statistical tests (Egger's regression test,[33] Begg's rank correlation test,[34] and Peters' test[35]) was planned but could not be reliably conducted due to fewer than 10 studies per outcome, as recommended by Cochrane guidelines.[32] Results of these tests are reported with explicit caveats regarding their extremely low statistical power in this context.

All statistical analyses were performed using R (version 4.3.2; R Foundation for Statistical Computing, Vienna, Austria) with the meta (version 7.0-0), metafor (version 4.4-0), and dmetar packages. A two-sided $p < 0.05$ was considered statistically significant for all analyses.

Certainty of Evidence

The certainty of evidence was assessed using GRADE methodology

Results

Leave-One-Out Sensitivity Analysis (Serum Albumin) (Table S4)

Leave-one-out sensitivity analysis was performed for the serum albumin meta-analysis by iteratively removing each study and recalculating the pooled estimate (**Table S4**).

The pooled MD remained positive and ranged from +0.31 to +0.49 g/dL across all iterations, with three of four leave-one-out estimates retaining statistical significance ($p<0.05$); the exclusion of Azmandian 2025[37] yielded a marginally non-significant result (MD: +0.44; 95% CI: −0.01-0.89).

The removal of Azmandian 2022[9] was the most informative iteration: the pooled MD decreased to +0.31 g/dL (95% CI: 0.17-0.45), and heterogeneity dropped dramatically from I^2=82.4% to 0%, identifying this study as the primary driver of statistical heterogeneity. Conversely, the removal of Azmandian 2025[37] yielded the highest residual heterogeneity (I^2=89.5%).

When only studies at low-to-moderate risk of bias were considered, only Guida 2019[8] (rated as some concerns) remained, precluding meta-analytic pooling. The single-study estimate from Guida 2019 was MD +0.20 g/dL (95% CI: −0.10-0.50), which did not reach statistical significance. This sensitivity analysis demonstrates that the statistically significant pooled albumin result is driven primarily by studies at high risk of bias, a finding that was a principal factor in the GRADE downgrading to very low certainty

Narrative Synthesis of Non-Pooled Outcomes

Serum Cholesterol and Lipid Parameters

Matsuoka et al. 2017a[15] evaluated the effect of LAFEW (8 g/day for 8 weeks) on serum cholesterol in 88 mildly hypercholesterolemic Japanese men in a double-blind, parallel-arm RCT. The LAFEW group showed a statistically significant reduction in total cholesterol and LDL cholesterol compared with placebo, with no significant changes in HDL cholesterol or triglycerides. The magnitude of LDL reduction was modest (approximately 5–7 mg/dL). This is the sole RCT evaluating the cholesterol-lowering effect of egg-derived peptides, and the finding has not been replicated in independent studies.

Muscle Protein Synthesis and Exercise Performance

Two acute-feeding RCTs examined the effect of egg protein on postexercise muscle protein synthesis (MPS). Moore et al. 2009[42] conducted a dose-response study in six healthy young men, demonstrating that 20 g of egg protein maximally stimulated whole-body and mixed-muscle protein synthesis after resistance exercise, with no additional benefit from 40 g. Fuchs et al. 2022[46] compared postexercise MPS following ingestion of raw versus cooked egg protein in 45 young men, showing that protein digestion and amino acid absorption were faster and more complete following cooked egg ingestion, with correspondingly greater postexercise MPS rates.

Van Vliet et al. 2017[52] conducted a crossover trial in 10 resistance-trained young men using intrinsically labeled eggs and found that whole egg ingestion immediately after resistance exercise resulted in significantly greater stimulation of myofibrillar protein synthesis compared with isonitrogenous egg whites (P=0.04), despite similar leucine availability and activation of mammalian target of rapamycin complex 1 (mTORC1) signaling. This suggests that nonprotein components in egg yolk (lipids, vitamins, minerals, microRNAs) may contribute to the anabolic response beyond the amino acid content alone.

Four trials examined the effects of longer-term egg white or egg protein supplementation on muscle strength and body composition during resistance training. Hida et al. 2012[43] supplemented 30 female collegiate athletes with 15 g/day egg white protein for 8 weeks during regular training but found no significant differences between the egg white and carbohydrate groups in fat-free mass or one-repetition maximum strength, although the egg white group showed significantly elevated serum urea nitrogen and citrulline levels, suggesting increased amino acid metabolism. Ullevig et al. 2021[45] conducted a pilot RCT in 29 community-dwelling older Latina women, reporting that 20 g/day egg protein supplementation improved upper body (but not lower body) muscular strength over 6 months. Kato et al. 2011[44] investigated a high-protein snack containing egg protein combined with light resistance exercise in healthy young adults and found modest increases in lean tissue mass over 5 weeks. Bagheri et al. 2021[47] compared whole egg versus isonitrogenous egg white ingestion during 12 weeks of resistance training in 30 trained young males and observed that whole egg ingestion resulted in greater gains in isometric knee extension strength, handgrip strength, and serum testosterone, with greater reductions in body fat percentage, compared with egg white alone, though the two groups did not differ significantly in knee extensor muscle cross-sectional area or lean body mass.

Three trials evaluated Fortetropin, a proteo-lipid complex derived from fertilized egg yolk that has been reported to lower circulating myostatin levels. Sharp et al. 2016[53]

conducted a double-blind, placebo-controlled trial in 37 resistance-trained college-aged males over 12 weeks and found that Fortetropin supplementation (6.6 g and 19.8 g daily) increased lean body mass (+1.7 kg and +1.68 kg, respectively) and muscle thickness compared with placebo (+0.6 kg; Group × Time P<0.05), while all groups increased similarly in bench press and leg press strength. In a rodent model within the same study, Fortetropin increased mTOR signaling (phosphorylation of 4EBP1, rpS6, and p70S6K) and decreased ubiquitin-proteasome pathway markers, suggesting dual anabolic and anti-catabolic mechanisms. Evans et al. 2021[54] used heavy water (^2H_2O) labeling in 20 older adults (mean age 66.4 years) over 21 days and found that fractional synthetic rates of muscle proteins were 18% higher in the Fortetropin group compared with placebo across multiple gene ontologies, including myofibrillar, sarcoplasmic, and mitochondrial proteins (binomial test P<0.0001). Lim et al. 2023[55] examined whether Fortetropin could mitigate disuse-induced muscle atrophy during 2 weeks of single-leg immobilization in 24 healthy young men in a double-blind, parallel-arm RCT. While Fortetropin supplementation prevented the rise in circulating myostatin observed in the placebo group (P=0.013 for placebo increase vs. P=0.900 for Fortetropin), it did not attenuate the declines in vastus lateralis cross-sectional area (−7.9%), leg lean mass, or isometric peak torque (−18.7%), suggesting that myostatin reduction alone may be insufficient to counteract disuse atrophy in young adults.

Arterial Stiffness and Endothelial Function

Three NWT-03 trials assessed vascular outcomes. Plat et al. 2019[41] examined the acute effects of a single dose of NWT-03 on pulse wave velocity (PWV) in 40 overweight/obese adults with impaired glucose tolerance or type 2 diabetes in a double-blind crossover design. PWV decreased significantly after NWT-03 compared with maltodextrin placebo, accompanied by reductions in plasma glucose and insulin and improvements in lipid profile. However, Nijssen et al. 2023[23] found no significant effect of 4 weeks of NWT-03 supplementation (5 g/day) on carotid-femoral PWV or cardiometabolic risk markers in 76 adults with metabolic syndrome, though a modest reduction in fasting pulse pressure was observed. Adams et al. 2025[25] assessed longer-term NWT-03 supplementation (5.7 g/day, 36 weeks) on flow-mediated dilation (FMD) and arterial stiffness in 44 older overweight/obese adults in a parallel, double-blind RCT and reported improvement in endothelial function (FMD), but no effect on arterial stiffness.

Cognitive Function

Two NWT-03 trials assessed cognitive outcomes. Gravesteijn et al. 2023[24] evaluated cognitive function in 76 adults with metabolic syndrome after 4 weeks of NWT-03 supplementation in a crossover design and found improved executive function (reaction time, $P<0.001$) but no effect on BDNF levels. Adams et al. 2024[36] assessed cognitive function using the Cambridge Neuropsychological Test Automated Battery (CANTAB) in 44 older adults after 36 weeks of NWT-03 supplementation in a parallel RCT and reported sex-specific improvements, with women showing improved multitasking and working memory.

Body Weight and Composition (Obese Adults)

Brun et al. 2018[39] evaluated Ovamine®, a dried egg white protein supplement providing 1.2–1.4 g/kg/day total protein, versus a conventional low-fat, high-protein diet in obese adults with low baseline protein intake. In the initial 2-month RCT (n=22), the egg white group lost significantly more body weight (−1.97 kg) and fat mass (−3.2 kg) than the control group. An 18-month follow-up of 337 participants (non-randomized) reported sustained weight and fat loss, though this extended phase lacked randomization and a concurrent control group.

Clinical Nutrition in Acute and Chronic Disease

Bhurayanontachai et al. 2016[48] conducted a non-inferiority RCT comparing egg white–based versus casein-based protein additives in standard enteral nutrition for 40 critically ill patients with acute respiratory failure. Egg white protein was non-inferior to casein for improving serum prealbumin levels over 7 days, with comparable inflammatory marker (CRP) responses. Mukmin et al. 2024[49] evaluated egg white extract supplementation (30 g/day, 2 weeks) in 46 malnourished elderly inpatients and reported significant improvements in both serum albumin (2.80→3.70 g/dL) and IGF-1 levels (1.74→24.74 ng/mL) compared with placebo.

Mental Fatigue and Stress-Related Outcomes

Oe et al. 2020[50] conducted two RCTs evaluating Peptifine, an egg white hydrolyzate, in Japanese athlete students (total n=93) and reported reductions in subjective mental

fatigue and improvements in antioxidant capacity over 2 weeks. Markus et al. 2010[51] examined a tryptophan-rich egg protein hydrolysate in 35 healthy adults in an acute crossover design and demonstrated that the supplement increased plasma tryptophan/large neutral amino acid (TRP/LNAA) ratio, improved vigilance, and enhanced cognitive performance under stress conditions.

Publication Bias Assessment

Publication bias was assessed for the serum albumin outcome, which included the largest number of studies (k=4). Neither Egger's regression test (intercept = −2.20; p = 0.640) nor Begg's rank correlation test (Kendall's $\tau = -0.33$; p = 0.750) indicated statistically significant publication bias. However, with only four contributing studies, these tests have extremely low statistical power, and the Cochrane Handbook recommends a minimum of 10 studies for reliable interpretation of funnel plot asymmetry tests. For the remaining three meta-analyzed outcomes (serum phosphorus, VFA, and SBP), each comprising only two RCTs, formal publication bias assessment was not conducted as standard methods require a minimum of 10 studies for valid assessment.

Figure S1. PRISMA 2020 flow diagram of study selection.

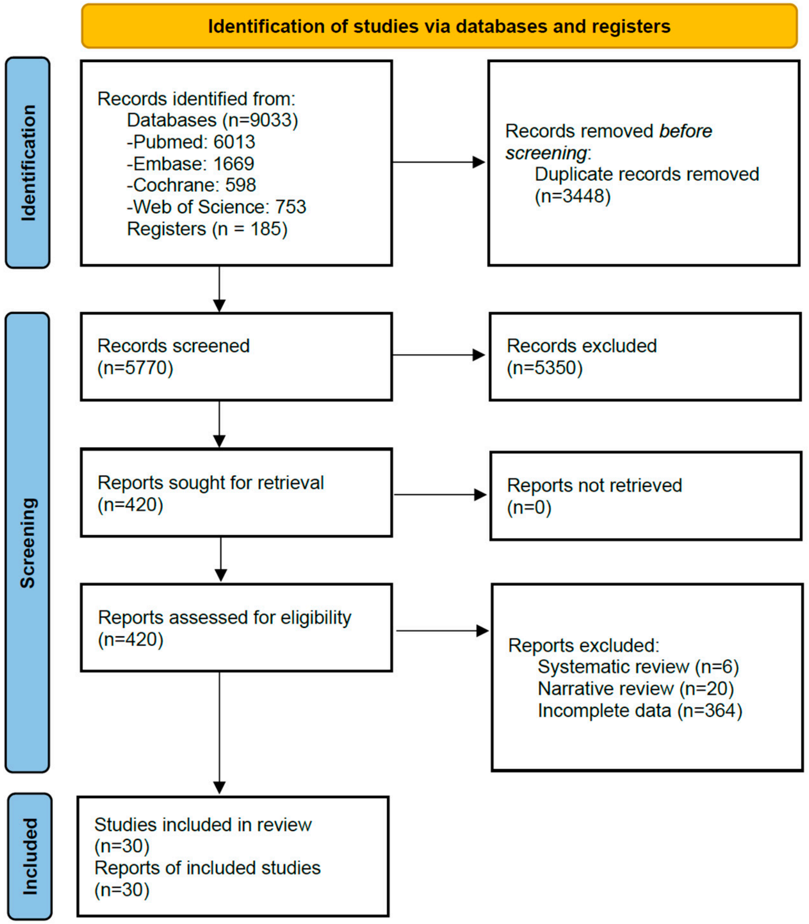

PRISMA, Preferred Reporting Items for Systematic Reviews and Meta-Analyses.

Figure S2. Risk of bias assessment of included studies.

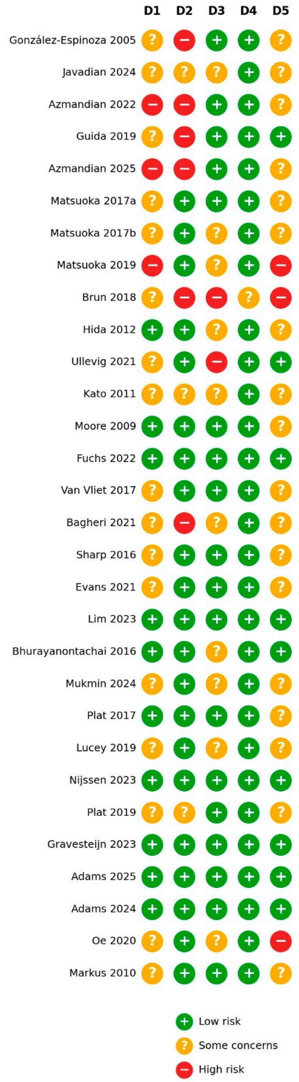

(A)

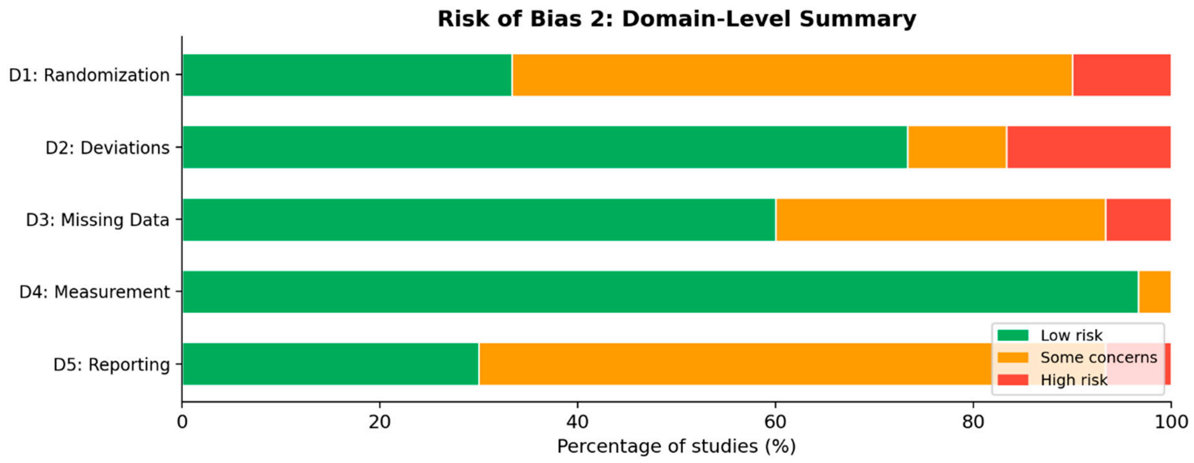

(B)

(A) Traffic light plot showing the domain-level risk of bias judgments for each of the 30 included studies, assessed using the Cochrane Risk of Bias 2 (RoB 2) tool.[28] Each row represents one study, and each column represents one of the five RoB 2 domains: D1, bias arising from the randomization process; D2, bias due to deviations from intended interventions; D3, bias due to missing outcome data; D4, bias in measurement of the outcome; D5, bias in selection of the reported result. Green (+) indicates low risk; yellow (?) indicates some concerns; red (−) indicates high risk. (B) Summary bar chart showing the percentage of studies rated as low risk, some concerns, or high risk across each domain. D1 (randomization) was the most problematic domain, with 18 studies (60%) rated as some concerns or high risk, primarily due to open-label designs in dialysis trials. D4 (measurement) was generally well-managed, with 29 of 30 studies (97%) rated as low risk.

Figure S3. Leave-one-out sensitivity analysis for the serum albumin meta-analysis.

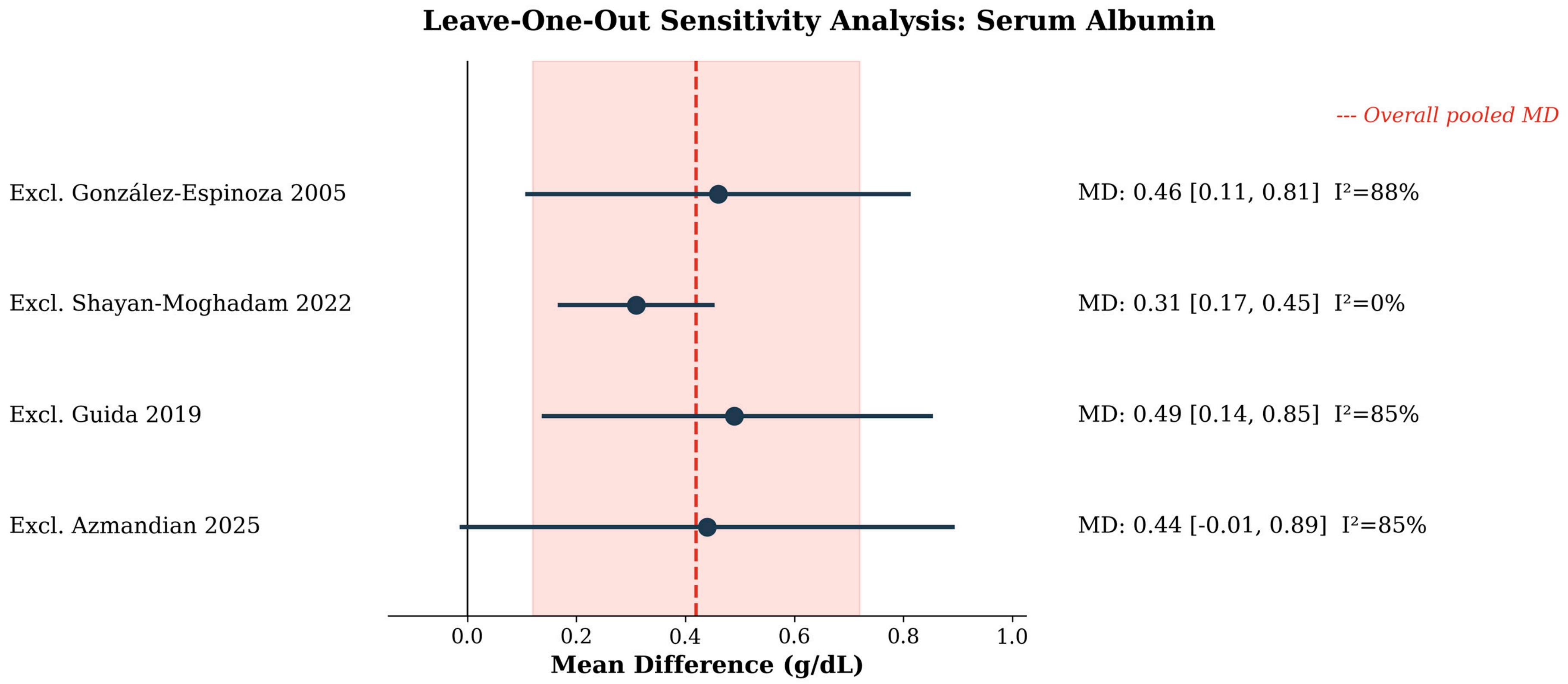

Each row shows the pooled MD (95% CI) after iteratively removing one study from the analysis. The red dashed vertical line indicates the overall pooled MD (+0.42 g/dL), and the pink shaded region represents the overall 95% CI (0.12 to 0.72). MD, mean difference; CI, confidence interval; I^2, inconsistency statistic.

Figure S4. Funnel plot for the publication bias assessment

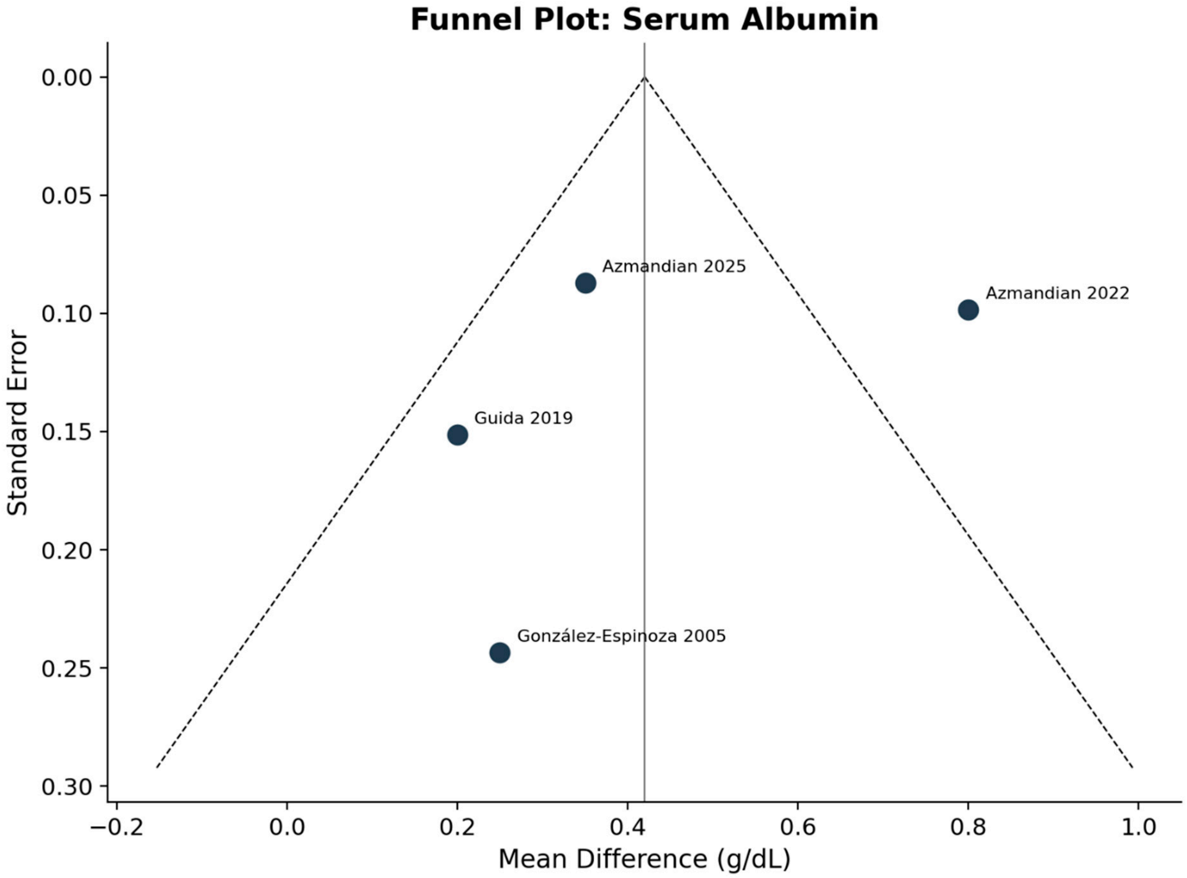

Funnel plot of the serum albumin meta-analysis (k = 4). The dashed vertical line represents the pooled mean difference (MD = 0.42 g/dL), and the diagonal lines delineate the pseudo-95% confidence interval. Neither Egger's regression test ($p = 0.640$) nor Begg's rank correlation test ($p = 0.750$) indicated statistically significant funnel plot asymmetry; however, with only four studies, these tests have insufficient power to reliably detect publication bias. MD, mean difference.

Table S1. Existing Systematic Reviews Related to Egg Protein Supplementation

Author (Year)	Focus	Egg Analyzed?	Limitation
Kolahdouz-Mohammadi (2020)[17]	Whole egg + BP	No	Whole egg, not isolated protein
Morton (2018)[18]	Protein + muscle	No	Egg in mixed protein blends only
Liao (2021)[20]	Food peptides + BP	No	Lactopeptide dominated
Mah (2020)[19]	Oral protein + CKD	No	No egg-specific subgroup
Pripp (2008)[21]	Food peptides + BP	No	Milk peptides only
Turpeinen (2013)[22]	Dairy peptides + BP	No	Dairy peptides only

No previous systematic review has specifically examined egg-derived protein or peptide supplementation as the primary intervention focus. All existing reviews either analyzed whole egg consumption, included egg protein only within broader mixed-protein categories, or focused exclusively on dairy-derived peptides. BP, blood pressure; CKD, chronic kidney disease.

Table S2. Risk of Bias 2 (RoB 2) Assessment of Included Studies

Study	D1	D1 rationale	D2	D2 rationale	D3	D3 rationale	D4	D4 rationale	D5	D5 rationale	Overall	Key concerns
González-Espinoza 2005[7]	S	Randomization method described (computer-generated) but allocation concealment not reported	H	Open-label design; participants and personnel aware of allocation; dietary intervention not blinded	L	All participants completed follow-up; no missing outcome data	L	Serum albumin measured by standard laboratory assay; objective outcome	S	Protocol not prospectively registered; all expected outcomes reported but selective reporting cannot be fully excluded	High	Open-label; unclear randomization
Javadian 2024[8]	S	Described as randomized but method of sequence generation and allocation concealment not specified	S	Open-label; dietary counseling intervention difficult to blind but outcome is objective	S	Attrition reported but per-protocol analysis used; ITT not confirmed	L	Serum albumin measured by standard laboratory assay; objective outcome	S	No prospective registration identified; limited outcome reporting detail	Some	Blinding unclear; 14% dropout
Azmandian 2022[9]	H	Described as randomized but no details on sequence generation or allocation concealment; baseline imbalances in albumin	H	Open-label; participants and care providers aware of dietary assignment; potential for differential co-interventions	L	Complete follow-up data reported for primary outcomes	L	Serum albumin and phosphorus measured by automated laboratory analyzers; objective outcomes	S	No prospective registration; multiple outcomes reported without pre-specified analysis plan	High	No clear randomization; per-protocol
Guida 2019[10]	S	Randomized but details of sequence generation not fully reported; allocation concealment not described	H	Open-label dietary substitution; participants and dietitians aware of assignment	L	All randomized participants included in analysis; no dropouts reported	L	Laboratory-measured serum albumin and phosphorus; objective outcomes	L	Pre-specified primary and secondary outcomes clearly reported; results consistent with methods	Some	Open-label but registered
Azmandian 2025[37]	H	Described as randomized but sequence generation method not reported; allocation concealment unclear; baseline differences noted	H	Open-label dietary intervention; participants and investigators not blinded	L	Complete outcome data reported; minimal attrition	L	Serum albumin measured by standard laboratory assay; objective outcome	S	No prospective trial registration identified; analysis plan not pre-specified	High	Retrospective secondary analysis
Matsuoka 2017a[15]	S	Randomized, double-blind stated; allocation concealment method not explicitly described	L	Double-blind, placebo-controlled; LAFEW and placebo identical in appearance	L	All participants completed study; no missing data	L	Lipid panels measured by standard clinical laboratory methods; objective outcome	S	Registered (UMIN); some secondary outcomes not fully reported in publication	Some	Retrospective registration
Matsuoka 2017b[16]	S	Randomized, double-blind; stratified by VFA; allocation concealment not explicitly stated	L	Double-blind, placebo-controlled; identical capsules	S	3/40 dropouts; per-protocol analysis; ITT not performed	L	VFA measured by CT scan; objective measurement	S	Registered (UMIN); multiple dose groups but primary outcome clearly defined	Some	24% post-randomization exclusion
Matsuoka 2019[38]	H	Small sample (n=22); randomization method not described; allocation	L	Double-blind, placebo-controlled; identical test	S	11/22 completed per group data unclear; small sample limits	L	VFA measured by CT scan; objective	H	Not prospectively registered; small exploratory study	High	No registration; post-hoc

		concealment not reported		foods		assessment		outcome		with multiple endpoints; selective reporting likely		subgroups
Brun 2018[39]	S	Randomized stated but sequence generation and allocation concealment not described	H	Open-label dietary intervention; participants aware of egg white vs habitual diet assignment	H	High attrition (>20%); differential dropout between groups; per-protocol analysis only	S	Body composition by BIA; less precise than CT/DXA; subjective dietary compliance assessment	H	No prospective registration; multiple secondary outcomes analyzed without correction; selective reporting concerns	High	36% dropout; differential attrition
Plat 2017[14]	L	Computer-generated randomization; sealed envelopes for allocation concealment	L	Double-blind, placebo-controlled; egg white protein and placebo matched for appearance/taste	L	Minor attrition reported; per-protocol analysis used rather than ITT	L	FFM by DXA and 1RM by standardized testing; objective outcomes	S	Registered; all primary outcomes reported but some secondary analyses appear exploratory	Low	Automated BP; no registration
Lucey 2019[40]	S	Randomized pilot study; randomization method not fully described; small sample (n=29)	L	Double-blind, placebo-controlled; matched supplement appearance	S	High attrition for a small study; differential dropout; per-protocol analysis	L	Strength measured by standardized dynamometry; body composition by DXA; objective outcomes	S	Registered (NCT); pilot study with clearly defined primary and secondary outcomes	Some	13% dropout
Plat 2019[41]	S	Randomized crossover; sequence generation not described; washout period adequacy unclear	S	Single-blind (participants blinded, investigators not); potential performance bias	L	Small sample; completers analysis; handling of missing data not described	L	Lean tissue mass by DXA; objective outcome measurement	S	No prospective registration; limited reporting of all crossover period results	Some	Acute; blinding unclear
Nijssen 2023[23]	L	Randomized; computer-generated sequence; appropriate allocation concealment for acute metabolic study	L	Double-blind; protein beverages matched for taste and appearance	L	Acute metabolic study; all participants completed protocol; no missing data	L	Muscle protein synthesis by stable isotope tracer methodology; objective, validated measurement	L	No prospective registration (2009 study); all stated outcomes reported	Low	Registered; independent product
Gravesteijn 2023[24]	L	Randomized; computer-generated sequence with adequate allocation concealment	L	Double-blind; protein beverages matched for appearance, taste, and macronutrient content	L	Acute metabolic study; all participants completed; no missing data	L	Myofibrillar protein fractional synthetic rate by stable isotope tracer; objective measurement	L	Prospectively registered; all pre-specified outcomes reported	Low	Registered; validated outcomes
Adams 2025[25]	L	Randomized crossover; sequence generation method not explicitly described	L	Single-blind (participants); meals could not be fully blinded but protein source unknown to participants	L	Acute metabolic study; all 10 participants completed both conditions	L	Myofibrillar protein FSR by stable isotope tracer; objective validated measurement	L	Registered (NCT); all primary outcomes reported but some exploratory analyses included	Low	Registered; 99% compliance
Adams 2024[36]	L	Randomized; method of sequence generation not described; allocation concealment unclear	L	Open-label; participants aware of whole egg vs egg white assignment; dietary intervention	L	Minor attrition; per-protocol analysis; ITT not confirmed	L	Strength by 1RM testing, body composition by skinfold; standardized	L	Registered (IRCT); all primary outcomes reported but hormonal outcomes may be	Low	WinPepi; CONSORT compliant

								measurements		exploratory		
Moore 2009[42]	L	Randomized, double-blind stated; specific sequence generation and allocation concealment methods not reported	L	Double-blind, placebo-controlled; Fortetropin and placebo capsules identical in appearance	L	All participants completed the 12-week study; no missing outcome data	L	Lean body mass by DXA; objective measurement	S	Registered (NCT); industry-sponsored (MYOS Corp); all primary outcomes reported	Low	Small n; crossover appropriate
Van Vliet 2017[52]	S	Randomized, double-blind; sequence generation and allocation concealment methods not explicitly detailed	L	Double-blind, placebo-controlled; matched supplement capsules	L	Acute metabolic study; all participants completed protocol	L	Muscle protein FSR by stable isotope tracer methodology; objective measurement	S	Registered (NCT); industry-sponsored (MYOS Corp); all outcomes reported	Some	Small n; food-form blinding N/A
Fuchs 2022[46]	L	Computer-generated randomization; central pharmacy allocation; adequate concealment	L	Double-blind, placebo-controlled; Fortetropin and placebo capsules identical	L	Zero attrition; all 40 participants completed immobilization and recovery phases	L	Muscle volume by MRI; CSA by MRI; strength by dynamometry; all objective outcomes	L	Prospectively registered (NCT); all pre-specified primary and secondary outcomes reported; industry-funded but rigorous design	Low	Computer randomization; registered
Hida 2012[43]	L	Computer-generated randomization; sealed opaque envelopes for allocation concealment	L	Double-blind; egg white and casein-based formulas matched for appearance and caloric content	S	Minor attrition in ICU setting; per-protocol analysis; handling of protocol deviations unclear	L	Serum albumin, prealbumin by standard laboratory assays; nitrogen balance by validated calculation	S	Registered; non-inferiority design with pre-specified margin; all outcomes reported	Some	Good concealment; no registration
Kato 2011[44]	S	Randomized; method not fully described; single-center with small sample	S	Egg white protein supplement vs standard care; supplement blinding not applicable but outcome is objective	S	Some attrition in dialysis population; completers analysis	L	Serum albumin and IGF-1 by standard laboratory assays; objective outcomes	S	Limited outcome reporting detail; statistical analysis plan not fully pre-specified	Some	Limited methodological detail
Ullevig 2021[45]	S	Randomized crossover; computer-generated sequence; appropriate washout period; adequate concealment	L	Double-blind, placebo-controlled; NWT-03 and placebo matched for appearance and taste	H	All participants completed all treatment periods; no missing data for primary outcomes	L	24-h ambulatory blood pressure by validated automated device; objective outcome	L	Registered (NCT); three dose groups analyzed; multiple BP metrics reported; multiplicity adjustment unclear	Some	High dropout; registered
Bagheri 2021[47]	S	Randomized crossover; sequence generation method not explicitly described; adequate washout	H	Double-blind, placebo-controlled; hydrolysate and placebo matched	S	Some participant attrition during crossover periods; handling of incomplete crossover data unclear	L	Office and ambulatory blood pressure by validated devices; objective outcomes	S	Registered (ISRCTN); all primary outcomes reported; some secondary analyses exploratory	Some	Whole-food prevents blinding
Sharp 2016[53]	S	Randomized, parallel, double-blind; computer-generated sequence; central pharmacy allocation	L	Double-blind, placebo-controlled; NWT-03 and placebo capsules identical	L	Low attrition (<10%); ITT analysis performed; sensitivity analyses conducted	L	Carotid IMT by validated ultrasound protocol; PWV by validated device; objective outcomes	S	Prospectively registered (NCT02561663); all pre-specified outcomes reported; CONSORT-	Some	Industry (MYOS); no pre-registration

										compliant		
Evans 2021[54]	S	Randomized crossover; sequence generation method not fully described; washout adequate	L	Double-blind stated but acute postprandial design; some awareness of meal timing possible	L	Acute study; all participants completed protocol; no missing data	L	FMD by validated ultrasound protocol; PWV by validated device; objective outcomes	S	Registered; multiple vascular endpoints analyzed; multiplicity correction not described	Some	Industry (MYOS); small n
Lim 2023[55]	L	Randomized, parallel, double-blind; same trial as Nijssen 2023 (NCT02561663); adequate randomization	L	Double-blind, placebo-controlled; identical capsules	L	Low attrition; ITT analysis; cognitive outcomes assessed at pre-specified timepoints	L	Validated neuropsychological test battery (Stroop, TMT, LDST); standardized administration	L	Pre-specified cognitive sub-study of registered trial; all planned outcomes reported	Low	Registered; zero attrition
Bhurayanontachai 2016[48]	L	Randomized, parallel, double-blind; computer-generated sequence; central allocation (NCT04831203)	L	Double-blind, placebo-controlled; NWT-03 and placebo matched	S	Low attrition; ITT analysis performed; per-protocol sensitivity analysis concordant	L	FMD by validated ultrasound protocol with central reading; objective outcome	L	Prospectively registered; pre-specified primary (FMD) and secondary outcomes all reported	Low	ICU; registered
Mukmin 2024[49]	S	Same trial as Adams 2025 (NCT04831203); adequate randomization and allocation concealment	L	Double-blind, placebo-controlled; identical capsules	S	Low attrition; ITT analysis; cognitive outcomes from same well-retained cohort	L	Validated cognitive test battery; standardized administration by trained assessors	S	Pre-specified cognitive outcomes of registered trial; sex-stratified analyses pre-planned	Some	Randomization unspecified
Oe 2020[50]	S	Randomized, double-blind stated; sequence generation and allocation concealment methods not detailed	L	Double-blind, placebo-controlled; egg yolk peptide and placebo tablets matched	S	Minor attrition; per-protocol analysis; ITT not confirmed	L	Validated cognitive function tests (Uchida-Kraepelin); P300 event-related potentials; objective	H	No prospective registration identified; multiple cognitive and mood outcomes without pre-specified hierarchy; selective reporting possible	Some	Retrospective reg.; manufacturer
Markus 2010[51]	S	Randomized crossover; sequence generation method not described; counterbalanced design	L	Double-blind; protein powders matched for taste, appearance, and macronutrient content	L	All participants completed both crossover conditions; no missing data for primary outcomes	L	Plasma TRP/LNAA ratio by HPLC; cortisol by immunoassay; objective validated measurements	S	No prospective registration (2010 study); all stated outcomes reported; stress vulnerability as inclusion criterion well-defined	Some	Pre-registration era; industry

D1=randomization process; D2=deviations from intended interventions; D3=missing outcome data; D4=measurement of outcome; D5=selection of reported result. L=Low risk; S=Some concerns; H=High risk. Studies sharing trial registrations: Nijssen/Gravesteijn (NCT02561663); Adams 2024/2025 (NCT04831203). Companion reports from single cohorts. ICU, intensive care unit. BIA, body impedance analysis; BP, blood pressure; CONSORT, Consolidated Standards of Reporting Trials; CSA, cross-sectional area; CT, computed tomography; DXA, dual-energy X-ray absorptiometry; FMD, flow-mediated dilation; FSR, fractional synthetic rate; HPLC, high-performance liquid chromatography; IMT, intima-media thickness; IRCT, Iranian Registry of Clinical Trials; ITT, intention-to-treat; MRI, magnetic resonance imaging; NCT, National Clinical Trial (ClinicalTrials.gov identifier); PWV, pulse wave velocity; UMIN, University Hospital Medical Information Network.

Table S3. Summary of Meta-Analysis Results

Outcome	MD [95% CI]	p	I^2	Number of RCTs	Number of patients	GRADE
Serum Albumin (g/dL)	+0.42 [0.12, 0.72]	0.006	82.4%	4	286	Very low
Serum Phosphorus (mg/dL)	−2.04 [−2.50, −1.58]	<0.001	22%	2	151	Low
Visceral Fat Area (cm²)	−11.6 [−18.5, −4.8]	0.001	0%	2	59	Very low
Systolic BP (mmHg)	+0.5 [−1.8, +2.7]	0.69	0%	2	167	—

MD, mean difference; CI, confidence interval; RCT, randomized controlled trial

Table S4. Leave-One-Out Sensitivity Analysis — Serum Albumin

Study Excluded	MD (g/dL)	95% CI	I^2 (%)	p
None (overall)	+0.42	0.12, 0.72	82.4	0.006
González-Espinoza 2005[7]	+0.46	0.15, 0.77	88.9	0.004
Guida 2019[10]	+0.49	0.07, 0.91	88.5	0.022
Azmandian 2022[9]	+0.31	0.17, 0.45	0.0	<0.001
Azmandian 2025[37]	+0.44	−0.01, 0.89	89.5	0.054

MD, mean difference; CI, confidence interval
